# Reconstructing Chromatic-Dispersion Relations and Predicting Refractive Indices Using Text Mining and Machine Learning

**DOI:** 10.1021/acs.jcim.2c00253

**Published:** 2022-05-19

**Authors:** Jiuyang Zhao, Jacqueline M. Cole

**Affiliations:** † Cavendish Laboratory, University of Cambridge, J. J. Thomson Avenue, Cambridge CB3 0HE, U.K.; ‡ ISIS Neutron and Muon Source, Rutherford Appleton Laboratory, Harwell Science and Innovation Campus, Didcot, Oxfordshire OX11 0QX, U.K.; § Department of Chemical Engineering and Biotechnology, University of Cambridge, West Cambridge Site, Philippa Fawcett Drive, Cambridge CB3 0AS, U.K.

## Abstract

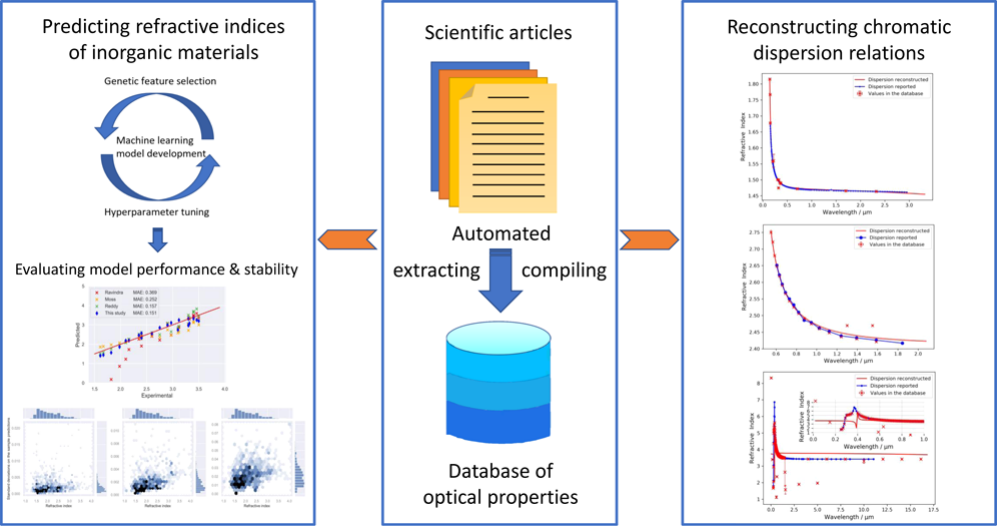

Predicting the properties of materials prior to their synthesis is of great significance in materials science. Optical materials exhibit a large number of interesting properties that make them useful in a wide range of applications, including optical glasses, optical fibers, and laser optics. In all of these applications, refraction and its chromatic dispersion can directly reflect the characteristics of the transmitted light and determine the practical utility of the material. We demonstrate the feasibility of reconstructing chromatic-dispersion relations of well-known optical materials by aggregating data over a large number of independent sources, which are contained within a material database of experimentally determined refractive indices and wavelength values. We also employ this database to develop a machine-learning platform that can predict refractive indices of compounds without needing to know the structure or other properties of a material of interest. We present a web-based application that enables users to build their customized machine-learning models; this will help the scientific community to conduct further research into the discovery of optical materials.

## 1 Introduction

One fast-developing topic in contemporary materials science is the high-throughput screening of prospective materials. The traditional combination of experimental work and computational modeling is in most cases time-consuming, expensive, and reliant on scientific intuition.^1,2^ Recently, the idea of using data science to model and design materials has received increasing attention. This has resulted in substantial improvements with respect to increased time efficiency and prediction accuracy of material properties.^2^ Using these techniques, material properties can be predicted and simulated prior to their synthesis. By constructing an open-source global repository of results from simulations and property models, researchers can find a better way to plan their experiments or computations without the need for repeating previously undertaken experiments.

Over the course of the past 30 years, studies trying to manifest the feasibility of modeling material properties by machine-learning technologies have emerged from intuition. For example, Pilania et al. trained kernel-ridge-regression algorithms on density functional theory (DFT) calculations of polymers and achieved an average accuracy of over 90% on validation sets that predict the atomization energy and the band gap of polymers.^3^ Ward et al.^4^ reported a broadly applicable feature set that contains 145 attributes to predict the properties of inorganic materials. They trained a fast decision-tree algorithm on DFT calculations to predict whether a composition can possibly form a metallic glass alloy. In 2018, Zhai et al. used 47 reported data of experimental Curie temperatures of perovskite materials to train several machine-learning models and achieved a mean percentage error of about 9%; they also proposed a perovskite material, La_0.66_Sr_0.3_Ba_0.04_MnO_3_,^5^ which was prospected to exhibit the highest predicted Curie temperature.

The refractive index is one of the most fundamental optical properties that describe how the speed of light travels within materials with respect to the speed of light in vacuum. Physically, the electromagnetic (EM) field inside the material is a superposition of the incident EM field and the stimulated EM field. The stimulated field arises from re-emissions of photons from electrons after multiple absorption mechanisms. However, the re-emitted photons might not be in phase with the incident photons. As a consequence, the superposition field is observed to be “slower” than the incident field. The refractive index of the material is a key parameter for device designs.^6^ The evaluation of refractive indices is of considerable significance for applications in integrated optic devices such as switches, filters, and modulators. Furthermore, knowing the refractive index and its chromatic dispersion is crucial for the evaluation of the suitability of a given material with nonlinear optical applications, for instance, their role in determining the phase-matching configurations for efficient sum-frequency generation. Therefore, many studies have been carried out to find an empirical formula that expresses the refractive index in terms of other physical properties.^7−11^ In general, the accuracy and generalizability of these relationships have been improved over time due to more materials being measured and the improvement of the measurement techniques. However, these estimations still suffer from their own shortcomings. Most of the estimations have favorable predictions only on a certain type of materials (semiconductors or Pb/SnTe alloys). These relationships require a knowledge of the band gap, which is not an easily accessible property for unseen or rarely used compounds. More importantly, the refractive index originates from multiple microscopic resonance mechanisms such as time-varying dipole moments of electrons, atoms, and other oscillators. Under the classical regime, the complex refractive index can be expressed in terms of resonance frequencies, ω_0_, of different oscillators^12^
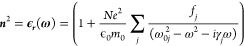
1where *f*_*j*_ is the oscillator strength representing the quantum-mechanical transition probability, *m*_0_ is the mass of the electron, and γ is the damping rate. As the band gap is a property of the electronic band transition, an expression of refractive indices in terms of band gaps will naturally miss the contributions of other oscillators.

Apart from theoretical modeling, efforts have also been made to model the refractive index by a data-science approach. Xuejing et al. collected refractive indices of 115 ionic liquids (ILs) and built an extreme-learning-machine (ELM) intelligence algorithm to predict the refractive index of ILs from molecular descriptors calculated by quantum chemistry.^13^ Haghighatlari et al. developed a deep neural network (DNN) to predict the refractive index of organic compounds based on 100,000 DFT calculations.^14^ They also employed topological and physicochemical features and molecular fingerprints as descriptors to construct numerical representations of molecules. Such modeling efforts either required the manual collection of data or the generation of data from DFT calculations, and the ranges of refractive indices in their data sets were relatively narrow: 1.35–1.60 in Xuejing’s study and 1.4–2.0 in Mojtaba’s study. These models also require the structure of the candidate material to be known and quantum-chemistry calculations of the structure to be available. However, since accurate quantum-chemistry calculations are computationally expensive, these constraints make it hard to screen a large set of novel candidate materials. The requirement of knowing the structure of the material forces scientists to screen only the materials whose structures have been reported in the literature.

This paper sets out to reveal the potential of an autogenerated database by performing a two-part downstream analysis. The first part of our analysis sets out to show the benefit of reconstructing chromatic-dispersion relations from a vast number of data sources. The second part of our analysis focuses on the development of a machine-learning model that has superior prediction power than empirical relations and can operate without the need for knowing other properties of the compound. The model is developed using source data from a material database of 49,076 experimental values of refractive indices for 6721 compounds.^15^ This database was autogenerated using the “chemistry-aware” natural-language-processing (NLP) toolkit, ChemDataExtractor.^16^ Relevant data from this database were used to explore three machine-learning models that are based on support-vector regression (SVR), random-forest regression (RFR), and Gaussian-process regression (GPR). Reference values of elemental properties^17^ were also used to aid the development of the machine-learning models. A web application is presented that allows the scientific community to query the refractive-index database and associated reference elemental properties with our machine-learning model to make their own refractive-index predictions for the compound of interest. We also demonstrate the feasibility of mapping chromatic-dispersion relations of compounds using the experimental database of refractive indices since it also contains their associated wavelengths. We begin by presenting the result of these mapping efforts.

## 2 Results and Discussion

### 2.1 Reconstruct Dispersion Relations of Different Types of Materials

Chromatic dispersion is a phenomenon whereby light beams of different optical frequencies travel at different velocities inside the material; this originates from different resonance strengths at different frequencies. As a real-life example, when sunlight is dispersed by droplets of water in the air, rainbows can be observed.

Material dispersion can be a desirable or undesirable effect in optical applications. For example, spectrometers are constructed from the advantage that light is dispersed when passing through glass prisms. However, chromatic dispersion is a serious consideration in long-haul optical fibers. Pulses always have finite spectral widths (bandwidth), and the dispersion will essentially stretch or flatten the initially sharply defined binary pulses of information. Thus, the large dependence of the pulse propagation on the chromatic dispersion requires knowing accurate chromatic-dispersion information about materials in optical-fiber applications. Refractive index and extinction coefficient are closely related to each other via the Kramers–Kronig relations.^12,18^ If the chromatic dispersion of a material is obtained, the absorption spectrum can then be calculated directly. This will help scientists to investigate crystal structures of materials and microscopic quantum-mechanical states of the molecules.

Owing to the costly nature of designing and conducting experiments, existing studies often focus on measuring refractive indices within a narrow range of wavelengths.^19,20^ The first contribution of our work is to show the ability of reconstructing chromatic-dispersion relations from refractive-index data that have been aggregated under different measurement wavelengths from a vast number of document sources. The complementary nature of these multiple source data can help researchers to ascertain information about dispersion relations that were not reported in the literature for compounds of interest to them. We begin with case studies of reconstructing chromatic-dispersion relations of several types of materials. The reconstruction was accomplished by fitting a second-order Sellmeier^21^ equation using the ordinary least-squares method with an L2 regularization on the fitting parameters. Their reconstructed dispersion relations are compared with the reported reference values taken from articles that are not present in our text-mining database of refractive indices and associated wavelengths.^15^ The refractive indices and dispersion relations of these materials have been widely reported, making these materials ideal candidates for evaluating our database and refractive-index-prediction toolkit. Moreover, the chromatic dispersion d*n*/dλ, the group-velocity dispersion (GVD), at the sodium D-line (589.6 nm), and the Abbe number of each candidate were calculated and compared with reported values. Overall, there are 138 compounds in our database that have more than 5 refractive-index data points of distinct measurement wavelengths, 78 compounds that have more than 8, and 59 compounds that have more than 10. When seeking a better quality of the reconstruction, we recommend using lower-order fitting functions for compounds that have no more than five data points of distinct wavelengths.

#### 2.1.1 Reconstructed Chromatic-Dispersion Relations of Glasses

Barium fluoride (BaF_2_) is an inorganic compound that occurs in nature as the rare mineral frankdicksonite.^22^ As a promising optical material with high density, it is commonly used to fabricate optical glasses, optical fibers, and laser generators. BaF_2_ is transparent from the ultraviolet to the infrared, and it is used in windows for infrared or ultraviolet spectroscopy. As one of the fastest scintillators, it is also used for the detection of X-rays, γ rays, or other high-energy particles.^23^

**Figure 1:**
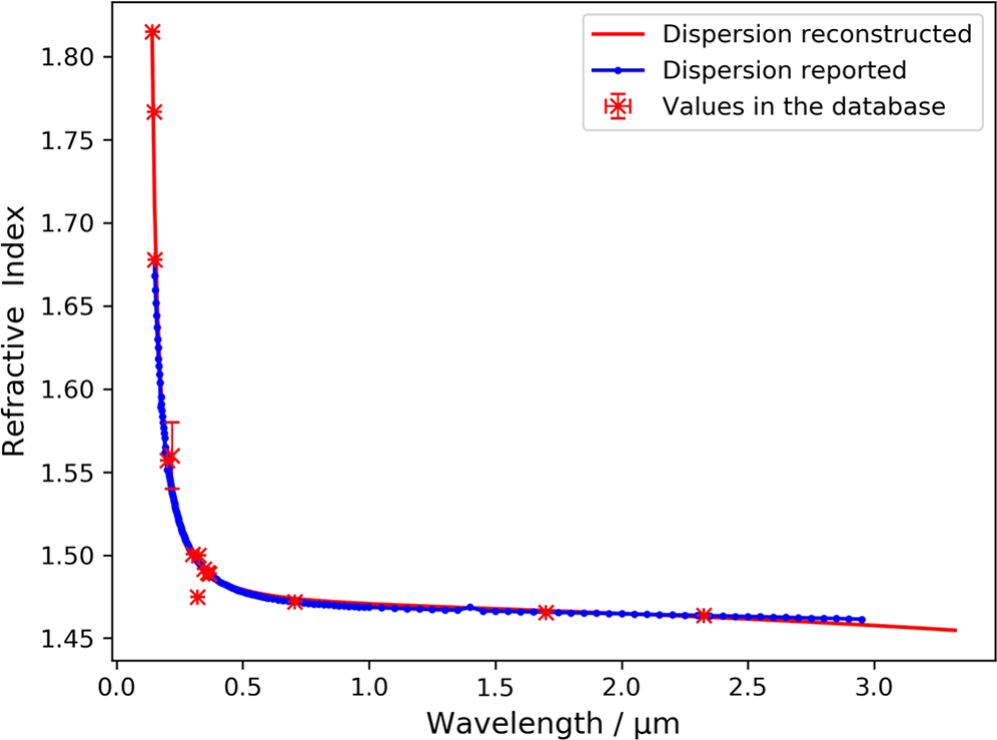
Reconstructed chromatic-dispersion relation of BaF_2_ alongside reported values^24^ that are not present in the corpus of scientific literature that was text-mined to afford our database.^15^ The red line indicates the reconstructed Sellmeier equation. Error bars on the red points show the standard deviation between values of individual measurements mined from different sources.

The corresponding dispersion relation of BaF_2_ that has been automatically reconstructed from our database sourced from the scientific literature^15^ is shown in Figure 1. Values in our database were obtained from five articles and covered a wide wavelength range. Error bars show the standard deviation between values of individual measurements mined for the same wavelength where multiple data exist. The reconstructed Sellmeier equation (eq 2) shows a high correlation with the generally reported trend. The denominators of our fitted equation also suggest that two of the absorption peaks of BaF_2_ are at 0.1172 and 30.17 μm
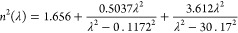
2

Chalcogenide glasses represent another important family of glasses within inorganic glasses. They have drawn increasing attention from both scientists and industry due to their excellent transmittance in the infrared region, a continuous shift of the optical absorption edge, and good mechanical properties.^20^ They have been used as infrared-transmitting materials in a wide range of optical devices such as far-infrared thermography systems, As–Se optical fibers, and acousto-optic modulators.^25^

**Figure 2:**
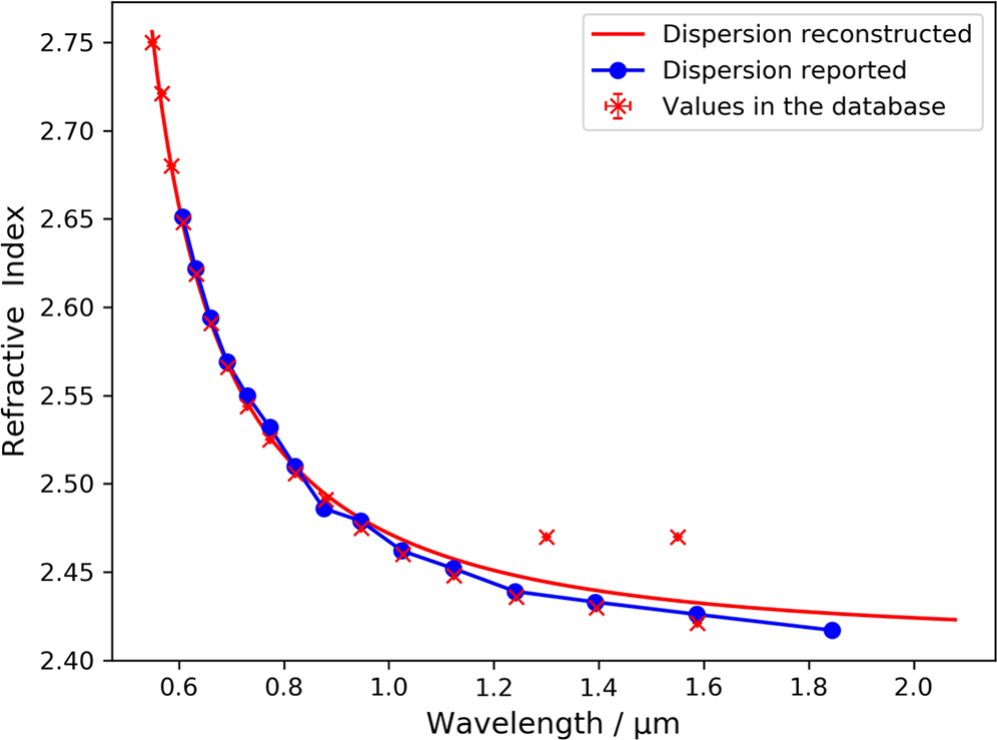
Reconstructed chromatic-dispersion relation of As_40_S_40_Se_20_ alongside reported values^20^ that are not present in the text-mined corpus. The red line indicates the reconstructed Sellmeier equation. Error bars on the red points show the standard deviation between values of individual measurements mined from different sources.

The wavelength dependence of refractive index for As_40_S_40_Se_20_, reconstructed from information in our NLP-generated database, is shown in Figure 2. The data points in our database were mined from three different articles. The reconstructed dispersion relation again shows a high correlation with the reported trend, although the fitted Sellmeier equation is slightly adrift by two outliers at near-infrared wavelengths. To the best of our knowledge, this is the first report of a fitted Sellmeier equation on As_40_S_40_Se_20_
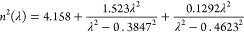
3

The absorption mechanism of inorganic glasses is mostly due to electronic-state transitions. Thus, inorganic glasses often display an absorption peak within the near-ultraviolet wavelength band. Our NLP-generated database has shown its potential in accurately reconstructing chromatic-dispersion relations of glasses within visible and near-infrared wavelengths, with additional functionalities of calculating group-velocity dispersion and Abbe number at given wavelengths. The fitted Sellmeier equation also provides the possibility of roughly estimating absorption peaks for the glasses. A more accurate estimation of the absorption peak may be achieved using a higher-order Sellmeier equation.

#### 2.1.2 Reconstructed Chromatic-Dispersion Relations of Oxides

As the second-most common oxide on the Earth, aluminum oxide (Al_2_O_3_) has been used widely in the material industry owing to its high hardness, excellent chemical stability, and high melting temperature. Al_2_O_3_ has also been found to present promising applications as an optical material. Owing to its low absorption among ultraviolet and visible bands, alumina films can be combined in multilayers with silicon dioxide (*n* = 1.48) for UV-laser applications.^26^ Amorphous Al_2_O_3_ also plays an important role in optical applications such as optical lenses and windows, antireflection coatings, and optical waveguides.^27^

**Figure 3:**
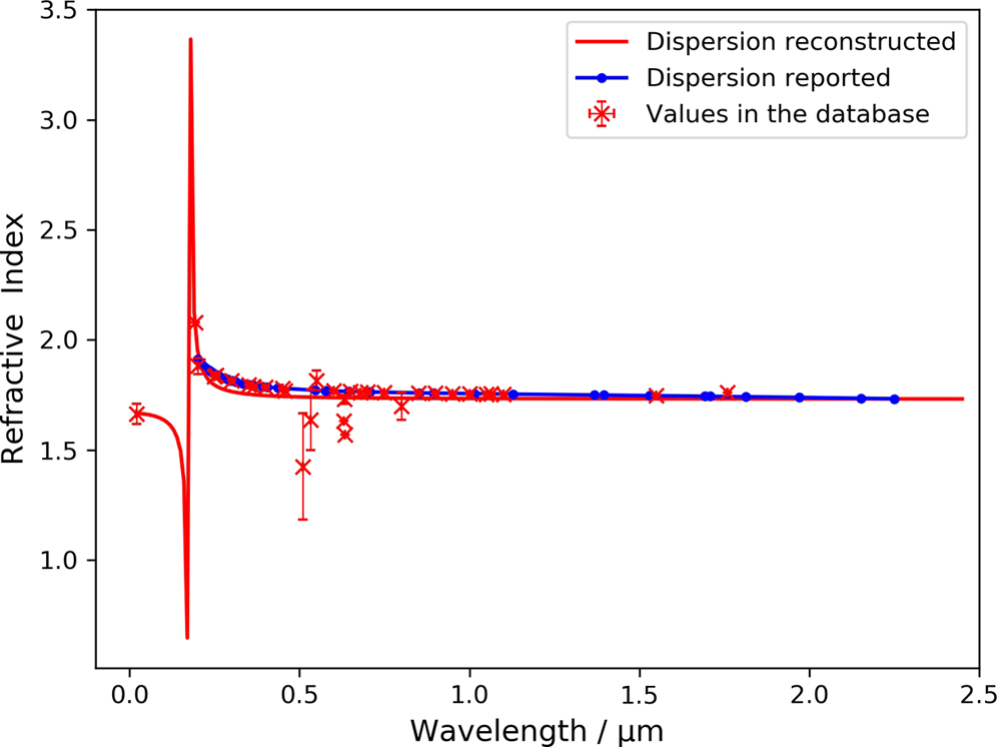
Reconstructed chromatic-dispersion relation of Al_2_O_3_ alongside reported values^28,29^ that are not present in the text-mined corpus.^15^ The red line indicates the reconstructed Sellmeier equation. Error bars on the red points show the standard deviation between values of individual measurements mined from different sources.

As it is a very popular material, there exist 56 refractive-index data with wavelength information of Al_2_O_3_ in our database, which were mined from 22 articles. The reconstructed Sellmeier equation is shown in Figure 3. The dispersion reconstruction shows a very similar trend to reported literature values. Some data points in our database are observed to deviate from literature values in the visible band, which is probably due to the fact that the refractive indices of Al_2_O_3_ are dependent on the degree of oxidation, the substrate temperature, and the crystal density achieved. Meanwhile, results from our NLP-generated database^15^ successfully predict the existence of an absorption peak below 300 nm.^26^ It is worth noting that there is only one value below the fitted peak. This brings possible sensitivity to the Sellmeier model. If this value is absent or incorrect, the fitting process might be affected. Users are recommended to pay extra attention to the data points that are near-resonance
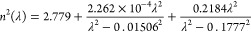
4

#### 2.1.3 Reconstructed Chromatic-Dispersion Relations of Organic Solvents

It has been widely reported that the solvent environment will affect the optical behavior of multiple synthetic products during the chemical synthetic process.^30−32^ As an example, acetone or propanone (chemical formula CH_3_COCH_3_) serves as an important organic solvent in its own right, in industry, at home, and in the laboratory. Many articles have reported the refractive indices of acetone when used as a solvent. Investigating the wavelength dependence of refractive indices of acetone will help the scientists better estimate the possible effect of acetone on the optical property of the product prior to the synthetic process
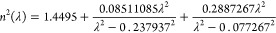
5

**Figure 4:**
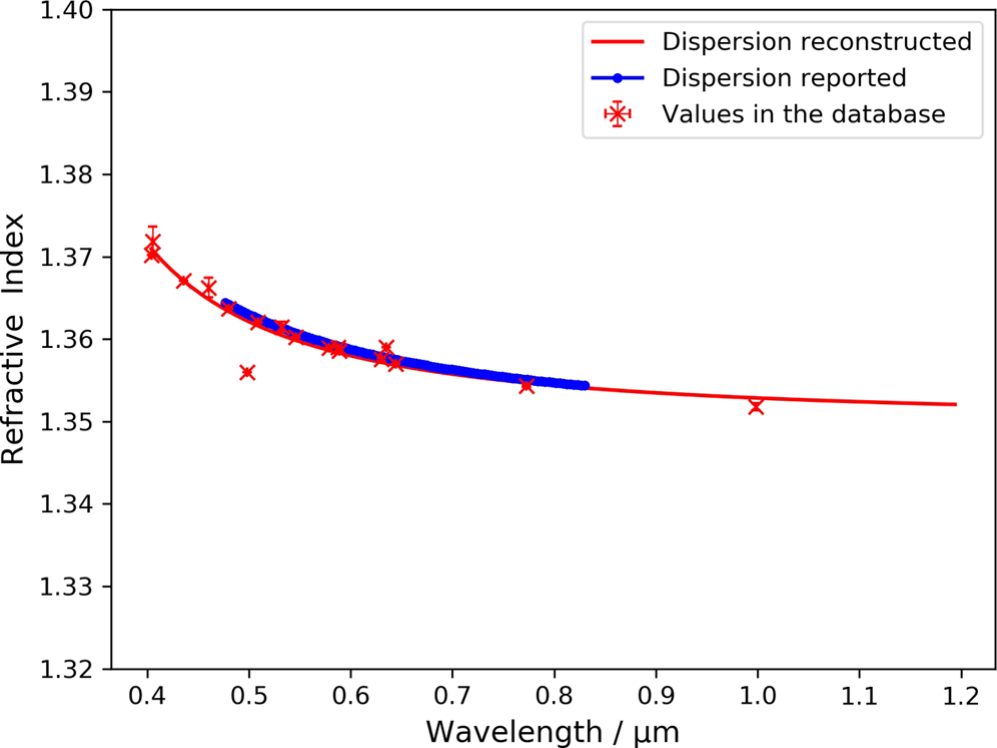
Reconstructed chromatic-dispersion relation of acetone alongside reported values^19^ that are not present in the NLP-generated database.^15^ The red line indicates the reconstructed Sellmeier equation. Error bars on the red points show the standard deviation between values of individual measurements mined from different sources.

The reconstructed dispersion (Figure 4) relation strongly agrees with the trend reported in the literature. The change in the refractive index of acetone as a function of wavelength is less than 2% across visible and near-infrared bands. This absorption-free behavior makes acetone a promising solvent in chemical synthesis under visible and near-infrared light environments.

#### 2.1.4 Reconstructed Chromatic-Dispersion Relations of Elements

**Figure 5:**
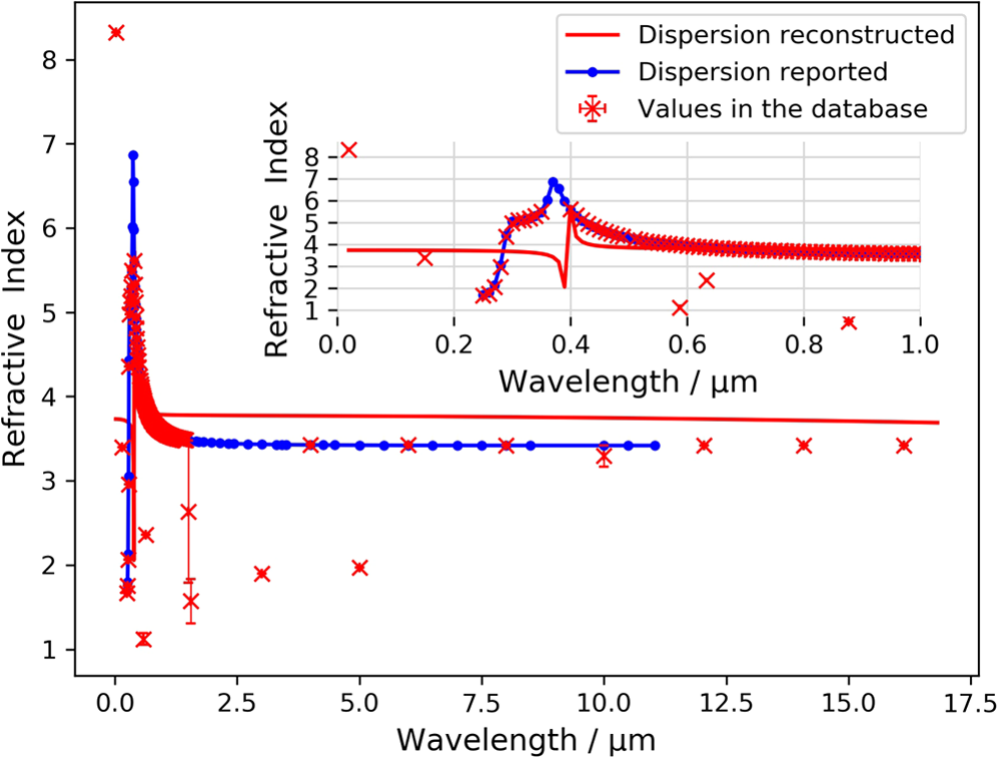
Reconstructed chromatic-dispersion relation of silicon alongside reported values^34,35^ that are not present in the NLP-generated database.^15^ The red line indicates the reconstructed Sellmeier equation. Error bars on the red points show the standard deviation between values of individual measurements mined from different sources.

The optical properties of crystalline semiconductors play significant roles in pure physics and materials-science research. Knowledge of parameters related to these properties, primarily for silicon and III–V semiconductors, has attracted great attention and received a high priority in microelectronics and optoelectronics since the establishment of these industries.^33^ The reported and reconstructed dispersion relations of silicon are shown in Figure 5.
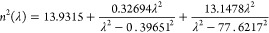
6

A total number of 153 records on silicon from 12 articles were filtered from our database with measurement wavelength information. The original values in our database strongly agree with the reported trend. Both the reference and reconstructed diagrams show a clear refractive-index peak at around 380 nm. This refractive-index peak results from enhanced band transitions of electrons within crystalline silicon. As the photon energy increases, it is not just the electrons that already have energies close to that of the band gap; the electrons within lower bands can also interact with the photon. Therefore, a larger number of electrons can interact with the photon, which results in an enhancement of photon absorption. It is worth noting that the inverse-hyperbolic nature of the Sellmeier model is less capable of giving an accurate estimation of the refractive index near the absorption peak, which can be observed from the compromised fit in the subplot of Figure 5, but it nonetheless gives a rough estimation of the location of the absorption peak.

#### 2.1.5 Calculations of Parameters Related to Chromatic-Dispersion Relations

In optics and lens design, the chromatic dispersion d*n*/dλ, GVD, and Abbe number are frequently used to characterize the dispersion of certain materials. To gain a better understanding of our database application toolkit for reconstructing dispersion relations, these parameters were calculated from the fitted Sellmeier equations and are presented in Table 1. Parameters of BaF_2_ and acetone were found to be in very good agreement with reported values as they are colorless crystals and transparent liquid under visible light. Parameters of Al_2_O_3_ were found to deviate within the same order of magnitude as reported values; this deviation stands to reason, as Al_2_O_3_ is not absorption-free within the measured wavelength ranges and the refractive indices of Al_2_O_3_ possess larger noise levels in our database. Parameters of silicon show a deviation from the literature result by 1 order of magnitude. This confirms the aforementioned fact that the fitting of the Sellmeier equations on silicon is less useful as a result of the complex shape across its absorption peak. To the best of our knowledge, this is the first report of these parameters of As_40_S_40_Se_20_.

**Table 1:** Predicted Chromatic Dispersion d*n*/dλ, Group-Velocity Dispersion (GVD) at 589.6 nm, Abbe Number Calculated Using the Equations Outlined in Methods, and the *p*-value of the Two-Sample Kolmogorov–Smirnov (KS) Test^a^

	chromatic dispersion (μm^–1^)		GVD (fs^2^/mm)		Abbe number		
compound	this study	reported	this study	reported	this study	reported	*p*-value
BaF_2_	–0.027	–0.029	47.20	54.95	92.23	81.78	0.986
As_40_S_40_Se_20_	–1.620	N/A	6350	N/A	3.343	N/A	0.953
Al_2_O_3_	–0.024	–0.055	49.12	91.17	156.3	72.31	0.876
Acetone	–0.032	–0.033	69.74	65.81	56.13	54.46	0.999
Silicon	–0.229	–2.300	499.1	N/A	45.68	N/A	1.8 × 10^–8^

a The reported values were obtained from Mikhail’s calculations^37^ based on the reported refractive-index literature of these chemicals.

Apart from the calculation of these empirical parameters, we performed a two-sample Kolmogorov–Smirnov (KS) test^36^ to quantitatively measure the goodness of the fittings, whereby the two samples are (1) raw data points taken from reported studies, i.e., the blue points in Figures 1–5, and (2) refractive indices predicted by the fitted Sellmeier equations at discrete wavelengths of the reported values. The null hypothesis is set to be that the samples are drawn from the same distribution, i.e., the ground truth of the chromatic dispersion relation. The alternative hypothesis is that they are drawn from two distinct distributions. *P*-values that report the results of these tests are presented in Table 1. The large p-values of the first four compounds indicate that the null hypothesis of these compounds cannot be rejected. The statistical significance of the fitted Sellmeier equations is thus shown to be sufficiently capable of representing the experimentally validated dispersion relations. Meanwhile, a small *p*-value of silicon (≪0.01) again confirms that the fitting result of silicon is less satisfactory.

### 2.2 Predicting Refractive Indices of Inorganic Materials

We have seen a highly accurate reconstruction of the chromatic dispersion relations. However, our database application toolkit is not only designed to perform these reconstructions but a distinct contribution of our work is that the text-mined refractive-index records are automatically paired with the elemental properties of their constituent elements. Using these features and a database with high diversity, we are able to construct physically interpretable machine-learning models of refractive indices and therefore perform generic refractive-index predictions. Details of the full list of descriptors used in this study and how they were constructed can be found in Table S4 and Section S2 of the Supporting Information.

#### 2.2.1 Model Development

Our machine-learning models were developed by deploying the pipeline in the Methods section. The support-vector regression (SVR) model performed best. Further details of results for other models can be found in the Supporting Information Section S3. All models were validated using two methods: predicting the refractive index of an external set of materials that are not presented in our database to estimate the accuracy of our models and “leave-one-out” cross-validation to compare the generalizability of our models. The performance of trained machine-learning models was compared against cognate results that stem from the use of empirical relationships to determine the refractive index of a compound that was developed by Moss,^7^ Ravindra et al.,^8^ and Reddy et al.,^9^ whereby
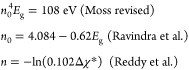
7

Unlike the traditional empirical approaches,^7−9^ our study demonstrated the potential of employing machine-learning techniques to find the most related physics-inspired descriptors that determine the refractive index. The SVR model suggests that the average column number, average row number, average number of *p* valence electrons, average electron affinity, average density, and the maximum difference in electronegativity of the constituent elements are the six most important features.

The average column number and average row number can directly reveal the information of the ionic radii of the constituent elements. The ionic radii are associated with the refractive index according to the Lorentz–Lorenz equation.^38^ The number or the configuration of valence electrons contributes to the refractive index via atomic electronegativity.^39−41^ For example, in TiO_2_, the electronic configuration of Ti is [1s^2^ 2s^2^ 2p^6^ 3s^2^ 3p^6^ 3d^2^ 4s^2^] and of O is [1s^2^ 2s^2^ 2p^4^]. The highest occupied molecular orbital (HOMO) is formed by the hybridization of 3d orbitals of titanium and 2p orbitals of oxygen. Meanwhile, the lowest unoccupied molecular orbital (LUMO) is made up of only pure 3d orbitals of titanium. This gives rise to a difference in the nature of the HOMO and LUMO called “dissimilar parity”.^42^ This dissimilarity will reduce the transition probability of the excited electron in the LUMO falling back to the HOMO, leading to a reduction of electron–hole pair recombination.^42^ The average electron-affinity descriptor is a measure of the capability of the constituent atoms to attract electrons. It can affect the optical refractive index by putting an impact on the ability to form instantaneous dipoles when atoms are exposed to external fields. The density of the compound is expected to be proportional to the average density of its constituent elements, and it will affect the refractive index through the number density of molecule per unit volume. The inclusion of the maximum difference in electronegativity between cations and anions, Δχ*, has a direct bearing on the concept of chemical bonding in nature.^9^ Meanwhile, the correlation between energy gaps and maximum differences in electronegativity has been enlightened by Duffy in a rough form of Δχ* = 0.2688*E*_g_.^43^ For materials that can be well described by the classical oscillator theory, Herve and Vandamme show that accurate results of the refractive index can be directly calculated from the energy gap.^11^ To this end, by virtue of the automated feature-selection algorithm, we have shown that our machine-learning model can be directly related back to the underlying theory, and it will provide a fundamental base for the generalizability of our model.

#### 2.2.2 Model Evaluation

We begin our model-evaluation process by predicting refractive indices of unseen data. Twenty-three refractive index data of 23 compounds that are not in our database^15^ (i.e., an out-of-sample test data set) were collected from the literature, mainly semiconductors, insulators, and oxides.^9^ The average absolute and percentage deviations from known values were calculated and are presented in Table 2. Our predictions are compared with the estimations obtained from the empirical relationships proposed by Ravindra,^8^ Moss,^7^ and Reddy,^9^ while there has been no report in the literature on the direct predictions of refractive index for this wide variety of materials using atomic features of their constituent elements.

**Table 2:** Refractive-Index Predictions Using the Machine-Learning and Feature-Selection Methods^a^

	refractive index, *n*						
material	SVR	RFR	GPR	Lit.	Ravindra	Moss	Reddy
CuI	2.468	2.462	2.077	2.35	2.26	2.38	2.517
BN	2.187	1.965	1.896	2.1	1.23	2.13	2.073
AIN	2.196	2.022	2.102	2.16	1.73	2.23	2.264
AlP	2.856	2.734	2.472	2.75	2.22	2.37	2.501
CuAlS_2_	2.329	2.424	2.490	2.4	1.91	2.28	2.346
CuAlSe_2_	2.549	2.607	2.571	2.6	2.41	2.44	2.606
CuInTe_2_	3.322	3.289	3.088	3.4	3.5	3.16	3.65
AgGaS_2_	2.568	2.553	2.482	2.4	2.41	2.44	2.606
AgGaTe_2_	3.309	3.039	3.038	3.3	3.4	3.05	3.504
AgInTe_2_	3.375	3.044	3.090	3.4	3.46	3.12	3.699
ZnSiP_2_	2.744	2.539	2.826	3.1	2.78	2.59	2.857
ZnGeAs_2_	3.200	3.365	3.152	3.5	3.37	3.01	3.459
ZnSnP_2_	2.978	2.997	2.751	2.9	3.05	2.75	3.092
CdGeP_2_	2.968	3.014	2.785	3.3	3.02	2.72	3.057
Ga_0.2_Al_0.8_As	3.109	3.129	3.050	2.97	2.48	2.46	2.649
Ga_0.6_Al_0.4_As	3.253	3.430	3.135	3.12	2.88	2.64	2.934
CdGe(P_0.2_As_0.8_)_2_	3.249	3.214	3.218	3.46	3.59	3.3	3.822
CdGe(P_0.6_As_0.4_)_2_	3.082	3.107	2.797	3.32	3.3	2.95	3.368
CsI	1.565	1.941	1.591	1.82	0.18	1.97	1.759
CsBr	1.447	1.643	1.505	1.67		1.89	1.584
CsCl	1.407	1.533	1.426	1.61		1.86	1.52
BaO	1.862	1.835	1.993	1.98	0.86	2.07	1.95
mean absolute error	0.151	0.168	0.210		0.370	0.252	0.158
mean percentage error	[HTML]333333 5.60%	[HTML]333333 6.19%	[HTML]333333 7.77%		12.40%	9.33%	5.82%

a A minimum mean absolute error (MAE) is achieved with support-vector regression (SVR) and genetic-algorithm (GA) feature selection. The estimations calculated from empirical relationships 7–9 are listed for a better comparison. Results from the full set of models, which were explored to predict these refractive indices, are provided in the Supporting Information (Table S8).

**Figure 6:**
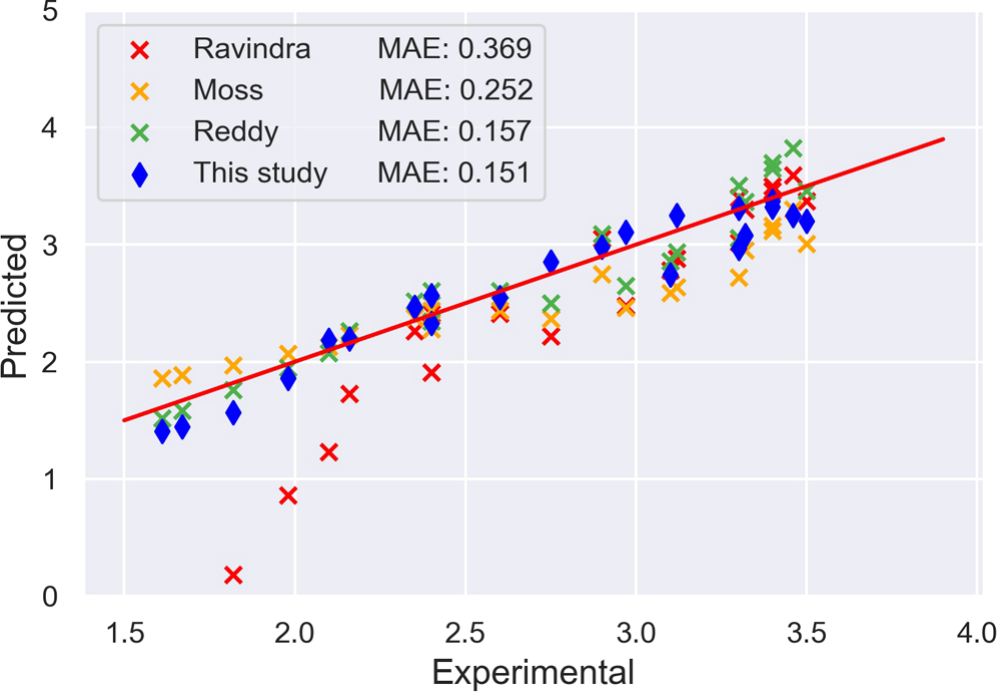
Predictions of refractive indices obtained from different methods versus known experimental values. The present method matches or improves the performance of the state-of-the-art empirical methods, while it does not rely on additional experimental measurements, such as the band gap, that are needed by the empirical methods. The red line indicates the relation *y* = *x*.

The prediction accuracy of the presented model is shown to match or beat empirical relationships^7−9^ for materials that possess a refractive index between 1.5 and 3.5. For the case of materials that possess one conduction band and one valence band such as aluminum phosphorus (AlP),^44^ Finkenrath^45^ has pointed out that the important factor of electronic transition is not that the band gap, *E*_g_, is expanded by the Fermi energy, *E*_F_, but rather that the decrease of ϵ_∞_ is caused by the deficit of all band states between *E*_F_ and −[*E*_g_ + (*m*_e_/*m*_h_)*E*_F_]. Our model is shown to have the potential of avoiding this shortcoming of estimating the refractive index from the band gap, with a deviation from the experimental value of 0.106 on AlP for our model compared with a 0.53 from Ravindra, a 0.38 from Moss, and a 0.249 from Reddy. Again, a considerable contribution of this work is to provide predictions of refractive indices without the need for knowing any other information such as band gaps or optical electronegativities, which will make our prediction fast and more applicable to inorganic compounds with unseen compositions. Figure 6 shows a graphical representation of the present approach, compared with Ravindra’s relationship, Moss’ relationship, and Reddy’s relationship.

**Figure 7:**
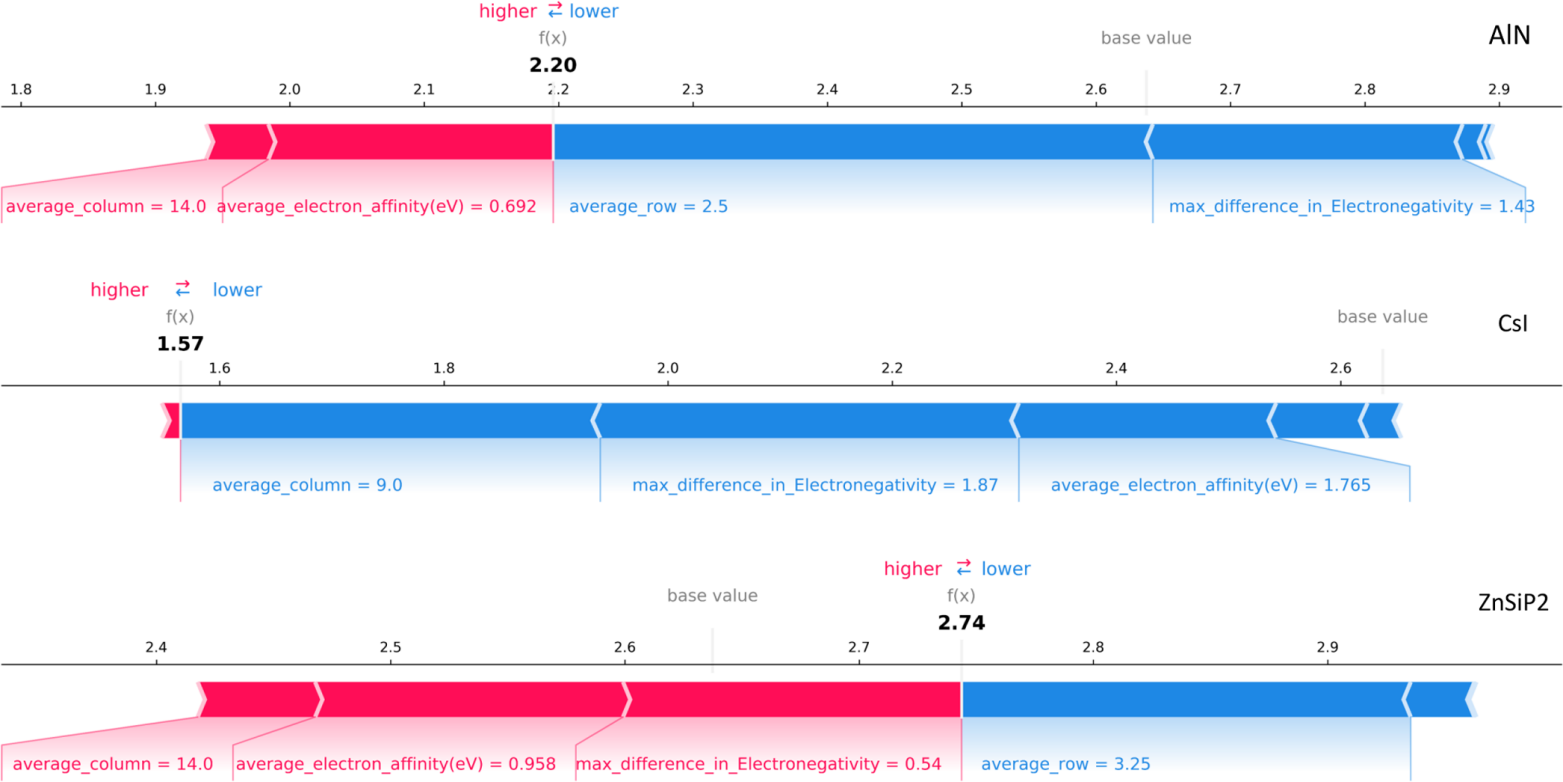
Visualization of the Shapley values of the SVR model of compounds AlN (top), CsI (middle), and ZnSiP_2_ (bottom). Bars in red indicate that this descriptor “pushes” to increase the prediction; bars in blue indicate that this descriptor “pulls” to decrease the prediction. The base value represents the value that would be predicted when no feature is known for the current output, i.e., the mean prediction of the test set. Only descriptors with top contributions are annotated.

To investigate how an individual substance depends on the selected descriptors, we additionally performed a local interpretation study on three randomly selected substances, AlN, CsI, and ZnSiP_2_. Shapley values^46^ of the SVR model and the selected descriptors are calculated and visualized in Figure 7. It is important to clarify two descriptors as a preface to the results of these case studies below. The “average row number” and “average column number” descriptors refer to the compositionally averaged position of each element of a compound within the periodic table, whose period number and group number define the row and column numbers described herein, respectively. For example, the average row number for the archetypal optical reference material, SiO_2_, is calculated according to the period number for Si (3) in the periodic table + 2 × the period number for O (2), all divided by 3 to yield the average row number, 2.33. Similarly, the average column number of SiO_2_ is calculated according to the group number of Si (16) in the periodic table + 2 × the group number of O (16), all divided by 3 to afford the average column number, 16.00. Thereby, these two descriptors are encodings of the periodic table that machine-learning algorithms can use to relate the physical property of a compound to a compositionally averaged elemental trend in the periodic table that pertains to this property.

The Shapley value is the average expected marginal contribution of one player, in our case, one descriptor, to the prediction, after all possible combinations have been considered. We now consider, in turn, the results of our three case studies. For AlN, two key descriptors that tend to pull its predicted refractive index toward a smaller value are the average row number (2.50) and the maximum difference in electronegativity, Δχ* (1.43), while an average electron affinity of 0.692 and an average column number of 14 tend to “push” its prediction toward a larger value. Similar roles of the average column number, the average electron affinity, and the average row number are observed in ZnSiP_2_. However, for ZnSiP_2_, a Δχ* of 0.54 tends to push its refractive index strongly toward a larger value instead of “pulling” it. Combining this finding with the observation that the strongest pulling tendency of the Δχ* descriptor comes from a Δχ* of 1.87 in the case of CsI, we suggest that our SVR model tends to assign a positive contribution to a smaller Δχ* value (≤1); this is comparable with the Δχ* value for a typical polar covalent bond (∼0.9). The results convey a distinct and quantitative model relationship between the polarizability of the compound and its composition, as one would expect for an optically active material. A similar trend is observed for the average electron affinity; this is reasonable as this property reflects the ability to form instantaneous dipoles when compounds are exposed to external fields. It is worth noting that the average column number (9) of CsI is calculated by averaging the column number of cesium (1) and that of iodine (17), while its Shapley value indicates that this descriptor tends to “pull” its prediction by ∼−0.3. The extent of this pulling effect is comparable with the deviation (−0.255) between the prediction and the ground-truth value of the refractive index of CsI. Without the consideration of the maximum difference in the column number, the average column number may lower the reliability of our result and bring a certain level of distortion to the final prediction. Overall, the local interpretation of AlN, CsI, and ZnSiP_2_ suggests that most of their selected descriptors contribute to the resultant prediction to a reasonable extent. The case of CsI also emphasizes the necessity of incorporating maximum-difference descriptors into model development.

**Figure 8:**
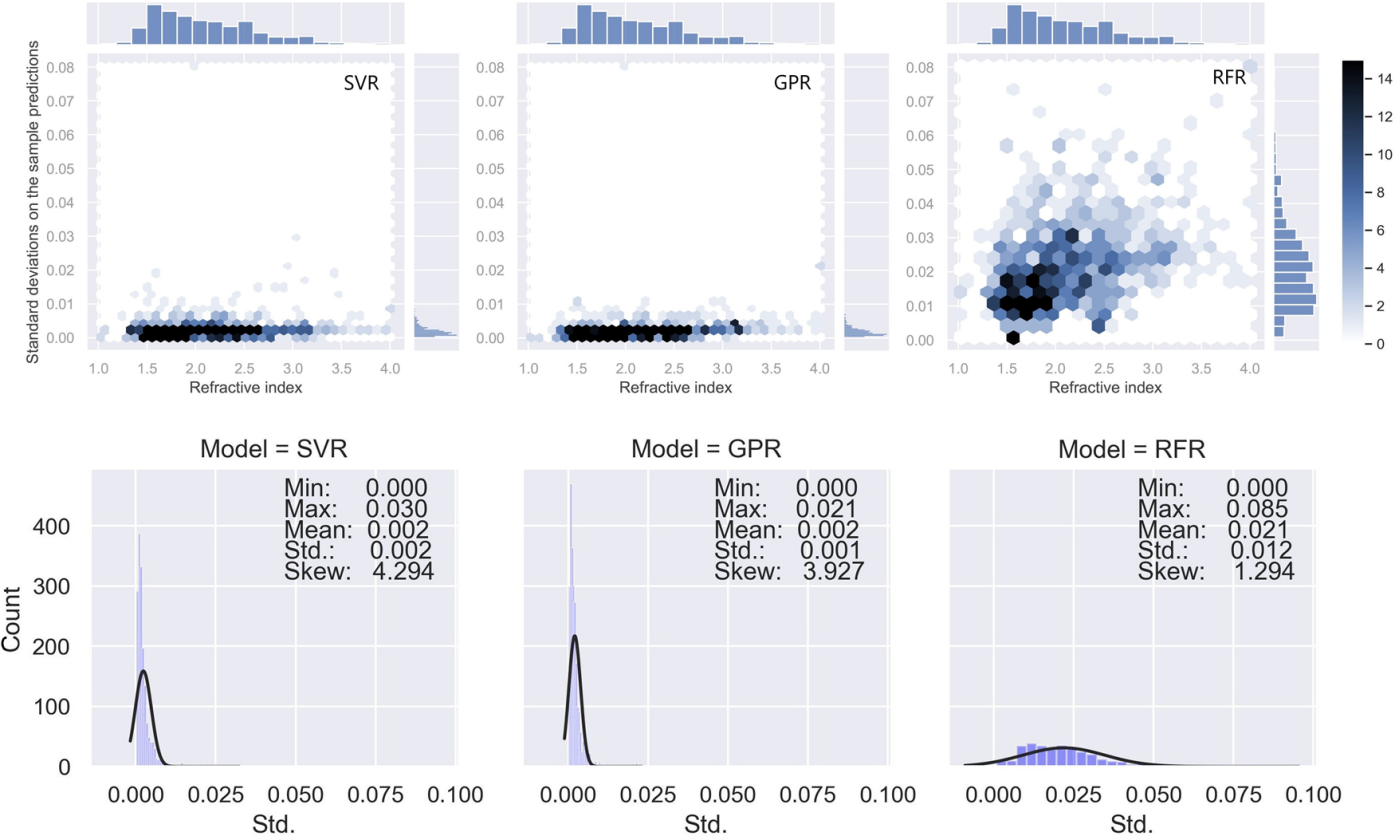
Top: hexagon plots that describe the joint probability distributions between the refractive-index value and the standard deviation of in-sample predicted values when the model hypothesis has been slightly changed. Bottom: corresponding histograms and statistics of these standard deviations. An original copy of this figure with more details, where the *x* and *y* scales are not fixed between plots in a line can be found in the Supporting Information (Figure S4).

Apart from its superior model accuracy, another key factor in describing the quality of our model is its generalizability. Within the framework of the learning theory, the algorithm stability has been employed as a useful tool to prove bounds on the generalization error of the model.^47,48^ The term “algorithm stability” refers to how much the prediction of the model changes when the training set is slightly modified. This idea is consistent with the metrics purposed by Huber of measuring the robustness of a statistical model.^49^ That is, (1) the model has a relatively high accuracy on the predicting target—which is also the most fundamental requirement of modern machine-learning models, (2) small variations in model hypothesis should only afford a small deviation in model performance, and (3) large variations in the model hypothesis should not bring a catastrophic effect on model performance. By employing the leave-one-out metrics described in the Methods section, the standard deviations of model predictions are visualized in the form of a hex plot and a histogram in Figure 8.

The histograms along the *x*-axis of these three plots are identical and indicate the distribution of refractive indices in our data set. The value on the *y*-axis represents the standard deviation of their predictions when one datum in the training set was omitted (see the Methods section). SVR and GPR show similar behavior in their standard deviations, where they both achieved a mean standard deviation of 0.002. This low level of variation suggests that the expected change of model prediction is exceedingly mild, approximately 0.1%, when the model hypothesis is changed slightly, i.e., one data point of the training set is omitted. The lowest standard-deviation level of the GPR suggests a success in preventing the model from overfitting by introducing a noise term, α, to the diagonal of its covariance matrix. However, the RFR was found to possess a significantly larger instability on its predictions, approximately 1 order of magnitude larger than that of the SVR and GPR. This behavior of the RFR might be a result of the fact that an algorithm built on tree-based predictors will have a larger potential of overfitting when there is an increased amount of noise in the sample.^50^ The problem of overfitting and the resultant model instability cannot be fully eliminated due to the nature of the algorithm itself and the existence of complex noise in our database.^15^ In conclusion, the SVR model with a genetic-algorithm-feature reduction shows the best predictive accuracy on unseen data and a promising stability when changing the model hypothesis. Thus, the SVR was considered to be our best model and set to be the default model in our prediction toolkit and web application.

Apart from empirical methods, efforts attempting to model refractive indices via machine-learning methods with a larger data set have appeared over recent years.^51−56^ These efforts have used data from two large databases of glass, INTERGLAD^57^ and SciGlass,^58^ which contain more than 300,000 refractive-index data of glasses. However, these databases are commercial databases (before 2019 for SciGlass) and they have been compiled manually.^56^ The refractive-index data presented in those studies lie within a range of 1.40–2.75. Compared with those efforts, the presented study is built on an open-source, autogenerated database from the scientific literature. Our model also covers a larger range (1.0–4.0) of refractive indices, and it is not limited to glassy materials. Although the predictive power of our model is slightly lower than those who used bigger data sets,^51−56^ we have revealed the potential and demonstrated the prospect of modeling the refractive index using an autogenerated database. This approach is intrinsically advantageous because more materials and properties can be added to our database by scripting methods. Thereby, the database can continue to grow, such that we will progressively be able to build predictive models with even greater detail and predictive power.

## 3 Conclusions

The pipeline and methodology presented in this study demonstrate the ability to fully integrate data that have been extracted from the scientific literature into machine-learning pipelines for material-property prediction. By aggregating data over a large number of independent sources, we were able to produce a large experimental database of certain material properties and negate the limitations of relying on small annotated data sets.

Overall, these case studies demonstrate that we can accurately reproduce chromatic-dispersion relations using the data mined from scientific literature and predict modest refractive indices for inorganic materials, using their elemental features as a basis. Compared with previous studies, our method could provide more accurate estimations than empirical calculations and cover a wider range of materials than computational modeling. Unlike estimations from empirical relationships,^7−9^ our model does not require any other information of materials such as band gaps or structural information but only elemental properties of their constituent elements. More importantly, the features automatically selected by the model were shown to provide profound physical insights according to current theories. The method exhibited in the study can be generalized in material design and controllable synthesis of other compounds, and it could further improve studies concerned with using machine learning to assist material design.

Looking ahead, we will continue to enhance our material discovery platform by adding new properties and new descriptors to the existing database, such as the dielectric constant and structural descriptors, as well as experimental parameters that are associated with each measurement. As the database continues to grow, and more properties are added, we will be able to build predictive models with even more details and generalizability, as the optical properties are intrinsically associated, and experimental parameters may play a significant role in determining material properties. An ultimate goal of our study is to predict and experimentally validate new classes of compounds for optical material applications.

## 4 Methods

The methodology for this work can be summarized in five stages: database creation, data standardization, chromatic-dispersion-relation reconstruction, refractive-index prediction, and the development of a web-based application.

### 4.1 Autogenerated Data Extraction and Database Creation

The data set used in this work is a database of refractive indices and dielectric constants for inorganic and organic compounds. A detailed description of this database and how it was constructed is given elsewhere.^15^ Thus, only a brief summary is provided herein. The data were automatically mined from text and tables contained within journal articles of the Royal Society of Chemistry, Elsevier, and Springer publishers, using a modified version of the state-of-the-art “chemical-aware” natural-language-processing (NLP) toolkit, ChemDataExtractor (version 2.0).^16^ A total number of 186,196 articles were sourced using the search query “refractive index” from the academic publishers mentioned above.

The mining procedure applied to these articles used a rule-based text parser, a semisupervised text parser,^59^ and a table parser,^16^ while the toolkit utilizes machine-learning processes, such as conditional random field model,^60^ to identify chemical-named entities and assign part-of-speech tags to words. This process yielded a set of 49,076 mutually consistent data records of 6,721 unique compounds. These data were collated in the database-management framework, MySQL, containing the chemical formula of a compound and its associated refractive index. Each entry was tagged with the digital object identifier (DOI), the authors, the journal name and the year of publication, etc., for the purpose of backvalidation. A detailed description of the format of the data record can be found in the Supporting Information (Table S1).

### 4.2 Data Standardization

The raw database generated by ChemDataExtractor^61^ is noisy and nonstandardized, as a certain fraction of records is false positive due to imperfection of the NLP process. To transfer the database into a usable data set for large-scale analysis and machine learning, an automated data-standardizing process was applied to remove improper entries and standardize the form of data records. This standardization process contains four stages:

- Duplicate refractive index unification.
- Conversion of inorganic chemical formulae to Hill notation.^62^
- Outlier value removal.
- Machine-learning descriptor construction.

**Figure 9:**
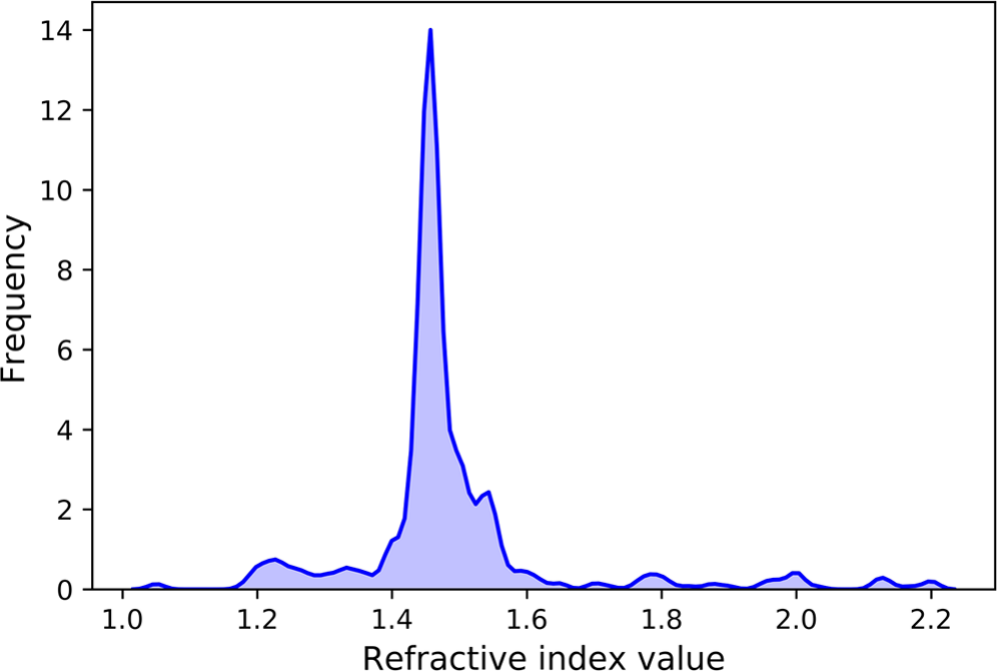
Kernel density distribution of records of SiO_2_ in our database.

It is often the case that one compound possesses multiplicate refractive indices mined from different sources in the raw database. A case in point is that SiO_2_ was found to have 948 records in the database as it is a very popular material in optical applications. We employed the idea that the likelihood of a record of being correct is proportional to the frequency that its value was mentioned in the literature. Thus, for each unique compound, a kernel density distribution (e.g., Figure 9) was fitted to the histogram of its refractive-index values, and the peak value of its kernel density distribution was taken as its unique refractive index value.

The conversion of inorganic chemical formulae to Hill notation^62^ used the National Cancer Institute’s Chemical Identifier Resolver (CIR) through their Python wrapper, CIRpy,^63^ to convert the inorganic chemical names into the Hill formula.^62^ Only compounds with valid Hill formulae were retained in the machine-learning data set. As the refractive index of a material becomes significantly larger when approaching its absorption peaks, only compounds with modest refractive indices between 1 and 4 (accounting for 95.3% of the total data) were retained in the data set. At last, the set of descriptors used in machine learning was automatically constructed for each compound. The set of descriptors contained purely elemental properties at the atomic level, which were sourced from reference tables.^17^ This information includes, but is not limited to, intrinsic properties such as atomic weight, electronic properties such as atomic electronegativity, and thermal properties such as enthalpy of fusion. A detailed list and legend of descriptors used in this study can be found in the Supporting Information (Table S2).

### 4.3 Reconstructing Chromatic Dispersion Relations

For gases, if we agree to stay away from resonances, the damping can be ignored, and the formula for the index of refraction can be simplified with the binomial expansion,^64^
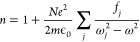
8For most substances, the natural frequencies ω_*i*_ are scattered all over the spectrum in a rather chaotic fashion. However, for transparent materials, the nearest significant resonances typically lie in the ultraviolet, so that ω < ω_*j*_. In that case, eq 7 takes the form^64^
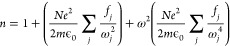
9Or in terms of the wavelength in vacuum (λ = 2π*c*/ω)
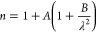
10This equation is known as Cauchy’s formula. In particular, Cauchy’s formula is only valid for regions of normal dispersion in the visible wavelength range. In the infrared, the equation becomes inaccurate, and it cannot represent regions of anomalous dispersion. The Sellmeier equation is a later development of Cauchy’s work that handles anomalously dispersive regions and more accurately models a material’s refractive index across the ultraviolet, visible, and infrared spectra. A two-term Sellmeier equation can be generally written as
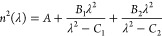
11The original data points in our database were fitted to this two-term Sellmeier equation to provide the reconstruction of the chromatic dispersion. The fitting was achieved using the “Nelder–Mead” minimization method provided by the Scikit-learn library^65^ together with an L2 regularization on fitting parameters. All original values in our database can be easily referenced back to their original articles by their DOIs. This permits backward validation and investigation of interesting or spurious values.

GVD is the phenomenon of the group velocity of light in a transparent medium depending on the optical frequency or wavelength. It was calculated as
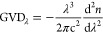
12The Abbe number, *V*_D_, is an early measure of the magnitude of chromatic dispersion introduced by Ernst Abbe
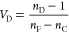
13The denominator is also called the principal dispersion. The Abbe number depends on the refractive indices at only three different wavelengths: *n*_F_ for 486.1 nm, *n*_D_ for 589.6 nm, and *n*_C_ for 656.3 nm.

### 4.4 Prediction and Feature-Selection Methods

In this work, we employed three machine-learning models: support-vector regression (SVR), Gaussian-process regression (GPR), and random-forest regression (RFR). These models were chosen among a wide range of machine-learning algorithms based on their model performances, model generalizability, and capability of interpretation. All of the prediction methods were implemented using the Scikit-learn Python library.^65^

SVR^66^ presents one of the most robust prediction methods based on the statistical learning framework or VC theory proposed by Vapnik and Chervonekis.^67^ The SVR problem can be formalized as
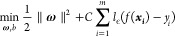
14where  is the model we want to learn, *y*_*i*_ is the target value, *C* is the regularization constant, and *l*_ϵ_ is the ϵ-insensitive loss function. By introducing slack variables ξ_*i*_ and ξ̂_*i*_, the Lagrange multipliers, , , , and , and the radial basis function kernel, *K*(***x***,***x***_***i***_), the solution of the Lagrange function of eq 14 can be expressed as
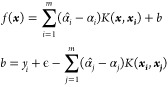
15GPR^68^ is a nonparametric model. It does not aim at finding an optimized weight, ω, to fit the pattern, but it follows a simple idea of “similar inputs yield similar output”. Since GPR inherits the mathematical foundation of Bayesian regression, it is able to provide a complete posterior for its predictions, i.e., not only the value of prediction but also its confidence interval. GPR assumes all data targets, i.e., *f*(*x*), belong to a Gaussian process
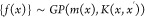
16where *m*(*x*) is the mean function of a Gaussian process, and *K*(*x*,*x*′) is the kernel function. In this work, we used the same kernel function that was used in SVR. After seeing training points, the regression problem then becomes solving conditional probability given the multivariate Gaussian distribution. The expected mean, μ_y*_, and standard deviation, Σ_y*_, of the predicted value y* have the following form
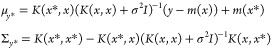
17RFR is a supervised learning algorithm based on decision trees, which uses ensemble learning method regression. Given a set of training samples *D* = {(*x*_1_,*y*_1_), (*x*_1_,*y*_1_), ...., (*x*_*m*_,*y*_*m*_)} and a set of features *A* = {*a*_1_, *a*_2_, ...., *a*_*d*_}, the generation of the decision tree is a recursion process: (i) generate the first node, (ii) split the sample set based on one selected feature a_*_ that will yield the best splitting result and omit that feature from the feature set, (iii) generate d branches for each feature *a*_*i*_ in the feature set, and (iv) for each branch, generate a node and perform steps (ii)–(iv) until the node reaches one of the following cases:

- All samples in the present node belong to the same class; no need to split again.
- The present feature set is empty, or all samples have the same value on all features; unable to split.
- The present node has no sample; unable to split.

Based on the decision tree-based estimator^69^ and bagging,^70^ random forest^71^ introduces a random choice of features into the decision-tree training process. This will enhance the generalization ability of the algorithm further from the increasing diversity between base estimators.

Feature selection and hyperparameter optimization were employed in the model-development process to find the most relevant descriptors, reduce model complexity, and improve model performance. For the feature selection, a genetic algorithm (GA) was found to outperform traditional methods such as selecting features based on a Pearson correlation coefficient or mutual information between the predictor and the target. A GA is a model-oriented stochastic method for function optimization based on the mechanics of natural genetics and biological evolution.^72^ It does not aim to identify shallow relationships between descriptors and a target; instead, it lets the model itself decide a most reasonable set of descriptors. An initial set of subsets of predictors, called a population, are created randomly. For each subset in the population, their performance is measured by a 10-fold cross-validation score. The subsets with the best performance are combined randomly to produce later generations that make up the next population, and it is expected that a better-performed subset will show up. To do so, individuals are selected and undergo cross-over (mimicking genetic reproduction) and also are subject to random mutations. This process is repeated over and over again until convergence is reached, i.e., the performance of the best subset does not change with generation.

**Figure 10:**
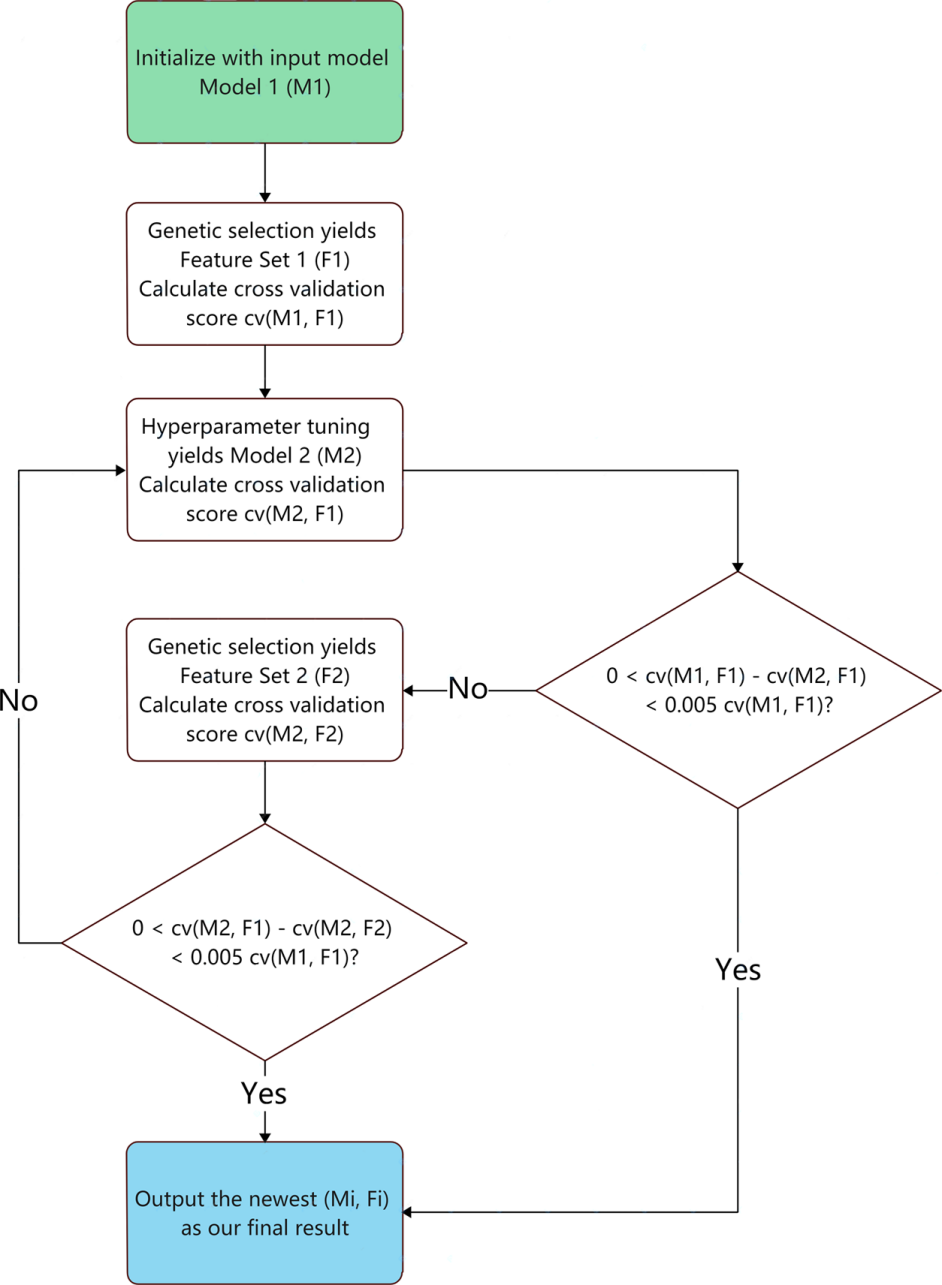
“F-H” pipeline that was designed to optimize both the feature set and the hyperparameter of the model.

For the hyperparameter optimization, the grid search method was used in this work. By setting the range and steps of hyperparameters, this method will loop through all combinations of hyperparameters within that range and release the best-performed hyperparameters. However, an obvious issue here is that the GA was evaluated based on the model tuned by hyperparameter optimization, and the hyperparameter optimization depends on the feature selected by GA. As the mathematical approach to this problem is arduous, we thus proposed the following “feature-hyperparameter” (F-H) pipeline (Figure 10) that performs feature selection and hyperparameter optimization iteratively to reach an optimum.

The general idea of this workflow is to find an optimized (feature, model) set by iterating the GA and grid search until the performance of (feature, model) becomes stable and converged. The evaluation metric of the model performance was chosen to be the mean absolute error of a 10-fold cross-validation. In a 10-fold cross-validation, the data set is split into 10 groups of equal size after shuffling. Each unique group is sequentially taken as the test set, while the remaining groups are taken as the training set. The average value of the 10 resulting mean absolute errors was used to score the model. The workflow is described in words as follows. A GA is first employed in the model with default hyperparameters, M_1_, and it generates the first selected feature set, F_1_. This is followed by the first grid search that will generate a new model, M_2_, based on the feature set F_1_. Now, the percentage difference, δ_*m1*_, between the 10-fold cross-validation score of (M_1_,F_1_) and (M_2_,F_1_) is calculated and stored in the cache. The GA is then applied again to the model M_2_ to generate a new selected feature set, F_2_. The percentage difference between the 10-fold cross-validation score of (M_2_,F_1_) and (M_2_,F_2_), δ_*f*1_, is then calculated and stored in the cache. This process is terminated if (1) the current score in the cache is the highest score and (2) the change in scores in the past three consecutive iterations is less than 0.1%.

**Figure 11:**
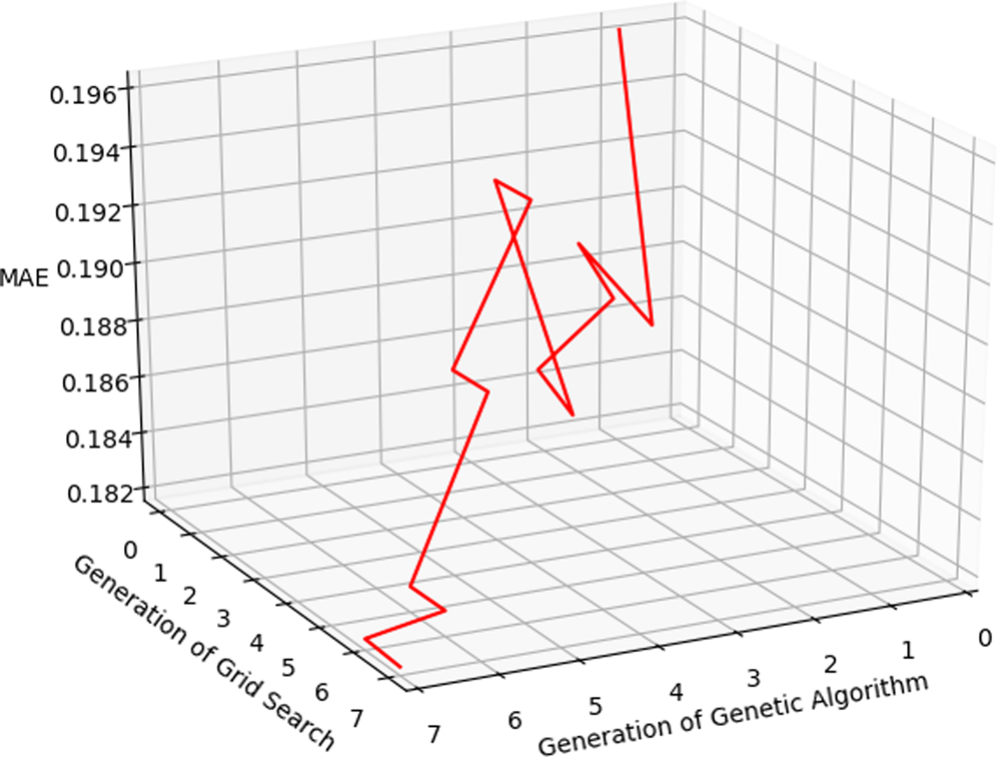
Variations of the model mean absolute error in the F-H iteration process. The algorithm first takes a step toward the *x*-direction for genetic feature selection and then takes another step toward the *y*-direction for hyperparameter optimization, eventually performing these steps alternately until convergence has been reached.

As an example, the support vector regression (SVR) is presented and visualized herein as our best model to demonstrate the model development process. Details of development results of other models can be found in the Supporting Information (Section S3). The feature-selection process and the hyperparameter optimization of the SVR were performed alternately according to the F-H pipeline to find a global minimum of MAE. This minimization process is shown in Figure 11.

**Figure 12:**
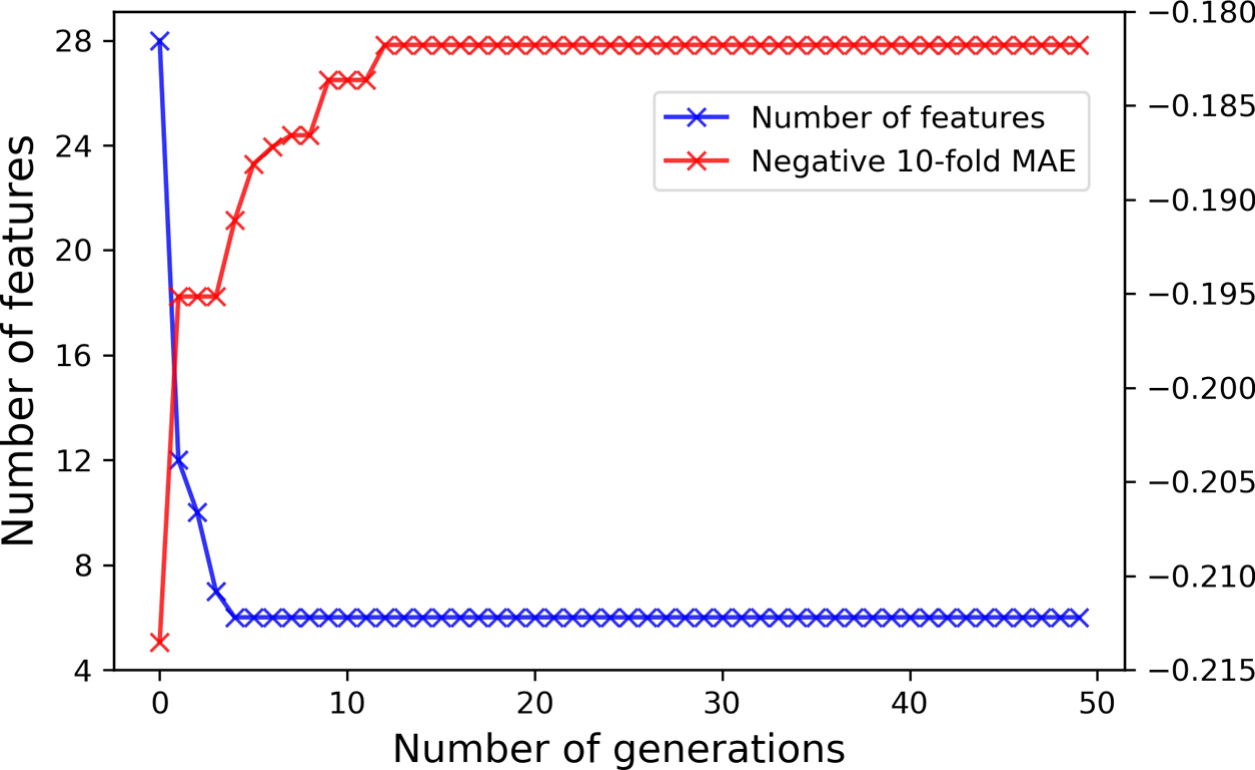
Reductions of the number of selected features and mean absolute errors in a 10-fold cross-validation versus generation in the genetic-algorithm-feature-selection process.

According to the “bias-variance dilemma”,^73^ a more complex model usually exhibits lower fitting errors on the training set but it may perform worse on the test set, i.e., overfitting. In general, the complexity of a machine-learning model is proportional to the number of features that it uses. The feature-reduction process is capable of both reducing the model complexity, shortening the training time, increasing the model generalizability, and removing features that are unrelated to targets. It can also reduce the undesirable effect of “multicollinearity”, i.e., a linear correlation between two descriptors in a multiple regression model, as it may bring a severe change to the prediction value if the input attribute is slightly changed. Analysis of the effect of the feature reduction on reducing multicollinearity can be found in the Supporting Information Section S4. The genetic algorithm feature selection process of SVR is visualized in Figure 12.

**Figure 13:**
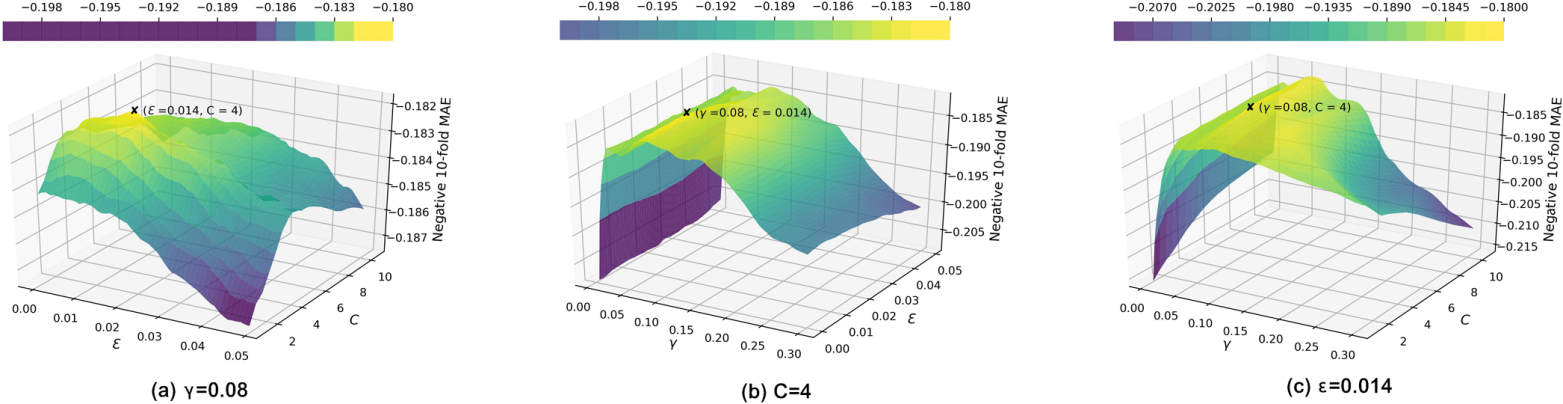
Hyperparameter optimization in SVR. For each plot, one parameter was kept fixed and the MAE variations on the other two parameters were visualized on a two-dimensional (2D) contour. The optimized values of these parameters are labeled by a cross on the plot.

For SVR, the controllable hyperparameters are *C*, γ, and ϵ. Parameter C determines the strength of the L2 regularization, and it was tuned from 0 to 10 with a step size of 1. Parameter γ is related to the σ value of the radial basis function (RBF) kernel by γ = 1/2σ^2^. If γ is too large, the RBF function will be too narrow, which may lead to overfitting. γ was tuned from 0 to 0.3 with a step size of 0.005. Parameter ϵ is a slack variable where the prediction with a residual less than ϵ was not counted in the loss function. ϵ was tuned from 0 to 0.05 with a step size of 0.001. The optimization process was performed in a three-dimensional space, and to give a better visualization, this process is illustrated in Figure 13: a plot of the variation of the MAE on two of these parameters while the remaining one is at its optimized value. Detailed information about the hyperparameter-optimization process for the other two models can be found in Section S3 of the Supporting Information.

It is worth noting that the repeated grid-search method for hyperparameter optimization is a brute-force method and it suffers from poor computational scalability. Consider a model that has *n* hyperparameters to be optimized and each hyperparameter has *k* options to evaluate in the grid search. The time complexity scales exponentially as O(*k*^*n*^). This compares with the time complexities in training the employed machine-learning models as follows: O(*m*^2^*d*) for SVR,^74^ O(*m*^2^d*m*_trees_) for RFR,^71^ and O(*m*^3^) for GPR,^68^ where *m* is the size of the training set, d denotes the number of features, and *m*_trees_ represents the number of trees in RFR, and the time complexity of a basic genetic algorithm O(*gps*) with *g* being the number of generations, *p* being the population size, and *s* being the size of the individual subset.^72^ The repeated grid search method thus becomes the bottleneck within computational scalability of our algorithm. Accordingly, we recommend using a hyperparameter optimization approach with better computational scalability such as random search^75^ or Bayesian optimization^76^ if the model that is used has more than five optimized hyperparameters.

### 4.5 Model Stability Evaluation Metrics

In our study, the algorithm stability is accessed by measuring the level of variation in the predictions of the samples in the following leave-one-out cross-validation:

1. Take the first data point in the data set, *x*_0_, as [test point].
2. Take the remaining *n* – 1 data points as [training set], where n is the total number of data in the data set.
3. Loop through each data point, *x*_*i*_, in the [training set]:
  - Take *x*_*i*_ point out of the [training set]. Now, the training set is of size *n* – 2.
  - Fit the model on the current training set (size *n* – 2).
  - Record the model prediction on the [test point] *x*_0_ and reset the model.
  - Put *x*_*i*_ back to the training set.
4. Calculate the standard deviation of the *n* – 1 predictions of *x*_0_.
5. Repeat this process for the second, third ... last data points.

At last, we obtain one standard-deviation value for each data point in our data set. Details of the comparative analysis between models are discussed in the Results and Discussion section.

### 4.6 Creating a Web-Based Application for Refractive-Index Prediction

A web-based platform (https://opticalmaterials.org) was created to embed the aforementioned machine-learning capabilities into the utility of our database^15^ so that the user can predict refractive indices of any compound of interest. Five machine-learning methods were employed: linear regression, ridge regression, support-vector regression, Gaussian-process regression, and random forest regression. The prediction tool has high flexibility in that the users can customize the feature-selection process; between GA, KBest, or using any combination of features they wish; or customize the hyperparameter-optimization process; using a grid search or any hyperparameters that they wish. A periodic table is embedded onto the website to help the user to quickly pick the combination of elements of interest. Details of these functionalities can be found on the documentation page of the website application.

## Supporting Information Available

Detailed descriptions of the format of the database record, features used for developing the predictive model, and the developing process of the GPR and the RFR models (PDF)

## Accession Codes

Python and its associated packages can be downloaded free of charge from https://www.python.org. The web application associated with this work is available at https://opticalmaterials.org. This contains all underpinning data, a data analysis user interface with an associated demo, usage documentation, and source code references with citing and licensing information.
