# Supplementary material for: Reconstructing Chromatic-Dispersion Relations and Predicting Refractive Indices Using Text Mining and Machine Learning
Source: J Chem Inf Model. 2022 May 19;62(11):2670–84. doi: 10.1021/acs.jcim.2c00253 (PMC9198980; doi:10.1021/acs.jcim.2c00253)
Supplement: Supplementary file 1 — ci2c00253_si_001.pdf [file ci2c00253_si_001.pdf]

Supporting Information: Reconstructing chromatic dispersion relations and predicting refractive indices using text mining and machine learning

Jiuyang Zhao[1] and Jacqueline M. Cole[1,2,3,*]

[1] Cavendish Laboratory, University of Cambridge, J. J. Thomson Avenue, Cambridge, CB3 0HE, U.K.
[2] ISIS Neutron and Muon Source, Rutherford Appleton Laboratory, Harwell Science and Innovation Campus, Didcot, Oxfordshire, OX11 0QX, U.K.
[3] Department of Chemical Engineering and Biotechnology, University of Cambridge, West Cambridge Site, Philippa Fawcett Drive, Cambridge, CB3 0AS, U.K.
[*] corresponding author(s): Jacqueline M. Cole (jmc61@cam.ac.uk)

Contents

1 Database Record Format **2**

2 Features Used for Developing the Predictive Models **2**

3 Development of the Gaussian process regression (GPR) and Random Forest regression (RFR) models **3**

3.1 Gaussian process regression . . . 3
3.2 Random Forest regression . . . 3
3.3 A complementary figure of Fig. 7. . . . 6

4 Testing the effect of the feature reduction on reducing multi-collinearity of the studied models. **6**

5 Results from the full set of models which were explored to predict the out-of-sample refractive indices **9**

1 Database Record Format

Table S1: Description of data records.

Data	Description	Data type
Property type	Optical property type	String
Compound	Chemical compound name	String
Normalised name	Normalised chemical name	List or String
Specifier	Material property specifiers	String
Raw value	Raw values presented in the article	String
Extracted value	Normalised values by ChemDataExtractor	Float
Extracted error[a]	Error extracted with the raw value	Float
Wavelength[b]	Measurement wavelength information	String
Dielectric loss[c]	Dielectric loss information	String
DOI	Source document DOI	String
Date	Source document publication date	String
Journal	Source document journal	String
Title	Source document title	String

[a] 'Extracted error' aim for cases such as '1.553 ± 0.05'.

[b] For refractive index data records only.

[c] For dielectric constant data records only.

2 Features Used for Developing the Predictive Models

The elemental features were then combined to calculate the compound-level features. We herein use Fe3O4 as an example to demonstrate this calculation, the current total number of features of this compound is illustrated in the bracket:

- Calculate the total number of atoms 3 + 4 = 7 (1)
- Calculate the total number of valence electrons 8 × 3 + 6 × 4 = 48 (2)
- Calculate the fraction of different valence electrons. (5) S electron fraction: (3 × 2 + 4 × 2) / 48 = 7/24 P electron fraction: (3 × 0 + 4 × 4) / 48 = 8/24 D electron fraction: (3 × 6 + 4 × 0) / 48 = 9/24
- Calculate the weighted average of polarizability, electronegativity, atomic weight, column, row, atomic number, covalent radii, number of s, number of p, number of d, electron affinity, heat capacity, density, enthalpy of fusion, melting point, boiling point, ionization energy. (23) For instance, average atomic weight = (3 × 55.845 + 4 × 15.999) / 7 = 33.076
- Calculate the maximum difference in atomic number, covalent radii, electronegativity, polarizability, ionization energy. (28) For example, Maximum difference in atomic number = (26 - 8) = 18

Table S2: Atomic and structural features used for developing the predictive models. Valence electrons, row number and column number were obtained directly from the periodic table. All other elemental properties were obtained from the CRC handbook [1].

Feature	Unit	Data Type
Atomic number	-	Integer
Atomic electric dipole polarizability	10^{-24} cm^3	Float
Number of s valence electron	-	Integer
Number of p valence electron	-	Integer
Number of d valence electron	-	Integer
Row number	-	Integer
Column number	-	Integer
Atomic weight, standard atomic weight	a.u.	Float
Electronegativity on the Pauling scale	eV	Float
Electron affinity	eV	Float
Heat capacity	$Jg^{-1}K^{-1}$	Float
Heat capacity	$Jmol^{-1}K^{-1}$	Float
Covalent radius	Å	Float
Density in liquid or solid phase	gcm^{-3}	Float
Enthalpy of fusion	$kJmol^{-1}$	Float
Melting point in	°C	Float
Boiling point in	°C	Float
Ionization energy	eV	Float

3 Development of the Gaussian process regression (GPR) and Random Forest regression (RFR) models

3.1 Gaussian process regression

The genetic algorithm feature selection process of GPR is visualised in Fig. S1.

3.2 Random Forest regression

The genetic algorithm feature selection process of RFR is visualised in Fig. S2.

For RFR, three hyperparameters (namely, estimators, max tree depth and min samples split) were optimized by using the grid search strategy. 'Estimator' determined the number of trees in the forest and was set from 1 to 100 with step 1. 'Max tree depth' indicated the maximum allowed depth of the tree and was set from 10 to 50 with step 1. 'Min samples split' controlled the minimum number of samples required to split an internal node and was set from 1 to 10 with step 1. In general, the ability of fitting is proportional to the number of trees, but the computing cost will also increase. While 'max tree depth' and 'min sample split' can help the model to reduce the risk of overfitting. The optimization process of RF was shown in Fig. S3 with the same manner as it of SVR.

The optimised hyperparameters of all three models are summarised and shown in Table S3.

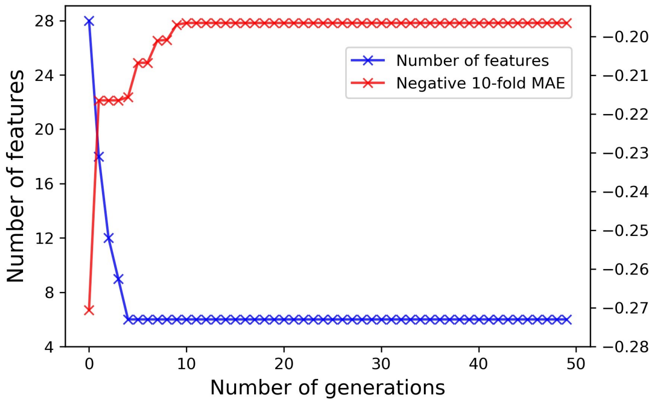

Figure S1: Reductions of the number of selected features and mean absolute errors of GPR in a 10-fold cross validation versus generation in the genetic algorithm feature selection process.

Table S3: optimization information of all three models including their ranges, steps and the best values that were found.

(a) Support Vector Regression (SVR)

Parameter	Range	Step	Optimised value
C	1 - 5	1	4
γ	0.005 - 0.300	0.005	0.080
ϵ	0.00 - 0.05	0.001	0.014

(b) Random Forest Regression (RFR)

Parameter	Range	Step	Optimised value
Number of estimators	10 - 100	1	69
Max tree depth	10 - 50	1	28
Min samples split	2 - 10	1	4

(c) Gaussian Process Regression (GPR)

Parameter	Range	Step	Optimised value
α	0.01 - 0.30	0.01	0.18

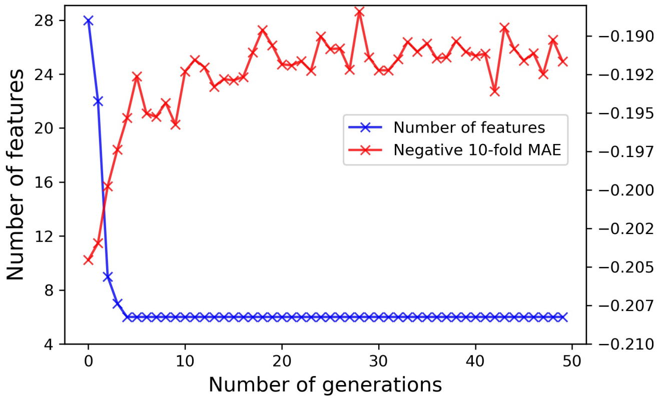

Figure S2: Reductions of the number of selected features and mean absolute errors of RFR in a 10-fold cross validation versus generation in the genetic algorithm feature selection process.

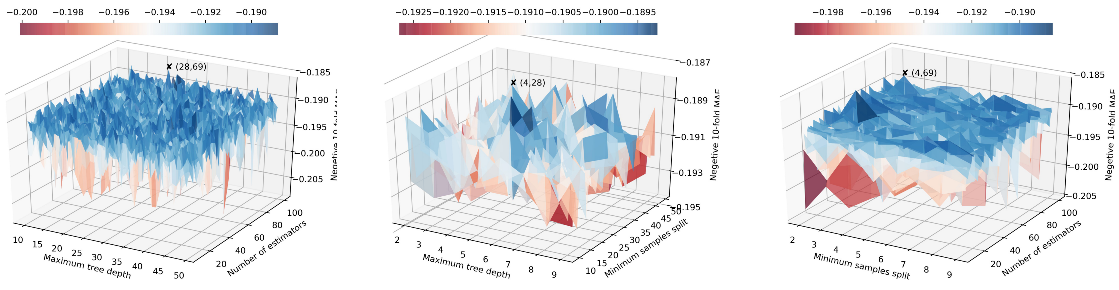

(a) split = 4 (b) estimator = 69 (c) depth = 28

Figure S3: Hyperparameter optimisation in RFR. For each plot, one parameter was kept fixed and the MAE variations on the other two parameters were visualized on a 2D contour. The optimised values of these parameters are labelled by a cross on the plot.

3.3 A complementary figure of Fig. 7.

A copy of the Fig. 7 in the paper with the x and y scales are not fixed between plots in a line is presented here to give a clearer vision on the details of these subplots.

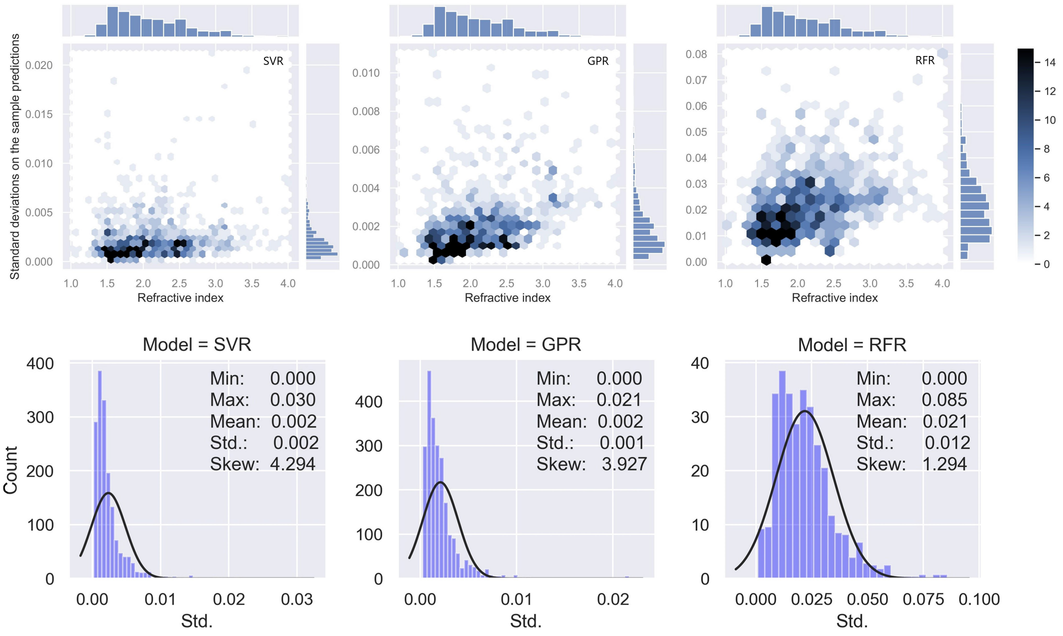

Figure S4: Top: Hexagon plots that describe the joint probability distributions between the refractive index value and the standard deviation of in-sample predicted values when the model hypothesis been slightly changed. Bottom: Corresponding histograms and statistics of these standard deviations.

4 Testing the effect of the feature reduction on reducing multi-collinearity of the studied models.

The multi-collinearity of the feature set before and after feature reductions was evaluated by the Variance inflation factor (VIF). VIF is the ratio of the variance of estimating some parameter in a model that includes multiple other parameters by the variance of a model constructed using only one term [2]. Considering the following linear regression model with parameters (features) X_1, X_2 ...:

$$Y = \omega_0 + \omega_1 \cdot X_1 + \omega_2 \cdot X_2 + ... \tag{1}$$

The VIF for the first variable X_1 is:

$$VIF_{X_1} = \frac{1}{1 - R^2_{X_1}} \tag{2}$$

where R_{X_1} comes from the coefficient of determination of the following linear regression model:

$$X_1 = \beta_0 + \beta_1 \cdot X_2 + \beta_2 \cdot X_3 + ... \quad (3)$$

In general, VIF describes the level of one parameter of being expressed as a linear combination of all other parameters. As seen in Eqn. 2, a VIF of 1 suggests this parameter is perfectly linearly independent of other parameters while a value of infinity suggests this parameter can be perfectly described by a linear combination of other parameters. It is suggested that a VIF > 10 is problematic in real-world applications [3]. We calculated the VIF values of the initial feature set (Table S4).

Table S4: A full list of the descriptors used in this study and their VIF values.

number_of_atoms	1.626821
average_Electronegativity	82.786077
average_polarizability	29.710668
average_ionization_energy(eV)	33.815531
average_atomic_weight	3666.757866
average_column	16.623545
average_row	633.012474
average_atomic_number	6010.846874
average_Cov_radii	299.068881
average_number_of_s	inf
average_number_of_p	inf
average_number_of_d	inf
average_electron_affinity(eV)	11.513058
average_cp/J g-1 K-1	10.453873
average_cp/J mol-1 K-1	2.855575
average_Density in g cm-3	50.357956
average_enthalpy_fusion(kJmol^-1)	5.102527
average_melting_point_y	9.914154
average_boiling_point	12.338436
total_valence_electron	inf
s_fraction	inf
p_fraction	inf
d_fraction	inf
max_difference_in_atomic_number	3.163697
max_difference_in_polarizability	13.136945
max_difference_in_ionization_energy(eV)	14.693904
max_difference_in_Cov_radii	28.418554
max_difference_in_Electronegativity	29.395822

Out of 28 features, only 5 have a VIF < 10. There also exist 7 features that have a VIF of infinity that suggests they can be perfected expressed as a linear combination of the other features. This indicates a serious collinearity problem existing in this feature set [3]. To check the effect of feature reduction on the collinearity problem of different models. We calculated the VIF values for three models after their feature reduction.

Table S5: VIF values of the features in the feature set selected by the Support vector regression (SVR) model.

average_column	5.004991
average_row	4.887693
average_number_of_p	4.756508
average_electron_affinity(eV)	2.261574
average_Density in g cm-3	6.649498
max_difference_in_Electronegativity	2.553016

Table S6: VIF values of the features in the feature set selected by the Random Forest Regression (RFR) model.

average_column	3.119100
average_Cov_radii	2.492628
average_electron_affinity(eV)	1.390069
average_Density in g cm-3	3.136290
max_difference_in_atomic_number	1.435825
max_difference_in_Electronegativity	3.747518

Table S7: VIF values of the features in the feature set selected by the Gaussian Process Regression (GPR) model.

average_Cov_radii	1.717044
average_electron_affinity(eV)	2.741454
average_Density in g cm-3	3.278511
s_fraction	3.434571
p_fraction	2.380062
max_difference_in_Electronegativity	2.359244

The VIF values for three feature sets after feature reduction are all < 10. It suggests that multi-collinearity problem is greatly mitigated after the feature reduction process.

5 Results from the full set of models which were explored to predict the out-of-sample refractive indices

Table S8: Results from the full set of models which were explored to predict the out-of-sample refractive indices. "Ridge" refers to the Ridge Regression model. "Linear" refers to the Linear Regression Model.

	Refractive Index, n					
Material	SVR	RFR	GPR	Ridge	Linear	Known
CuI	2.468	2.462	2.077	2.695	2.696	2.35
BN	2.186	1.965	1.896	2.117	2.117	2.10
AlN	2.196	2.022	2.102	2.296	2.296	2.16
AlP	2.856	2.734	2.472	2.546	2.547	2.75
CuAlS2	2.329	2.424	2.490	2.281	2.280	2.4
CuAlSe2	2.549	2.607	2.571	2.517	2.518	2.6
CuInTe2	3.322	3.289	3.088	3.249	3.249	3.4
AgGaS2	2.568	2.553	2.482	2.647	2.647	2.4
AgGaTe2	3.310	3.039	3.038	3.264	3.263	3.3
AgInTe2	3.375	3.044	3.090	3.381	3.380	3.4
ZnSiP2	2.744	2.539	2.826	2.448	2.448	3.1
ZnGeAs2	3.200	3.365	3.152	2.907	2.908	3.5
ZnSnP2	2.978	2.997	2.751	2.779	2.779	2.9
CdGeP2	2.968	3.014	2.785	2.770	2.771	3.3
Ga0.2Al0.8As	3.109	3.129	3.050	2.887	2.887	2.97
Ga0.6Al0.4As	3.253	3.430	3.135	3.008	3.008	3.12
CdGe(P0.2As0.8)2	3.249	3.214	3.218	2.984	2.985	3.46
CdGe(P0.6As0.4)2	3.082	3.107	2.797	2.877	2.878	3.32
CsI	1.565	1.941	1.591	2.087	2.087	1.82
CsBr	1.447	1.643	1.505	1.792	1.792	1.67
CsCl	1.407	1.533	1.426	1.501	1.501	1.61
BaO	1.863	1.835	1.993	2.024	2.024	1.98
Mean absolute error	0.151	0.168	0.210	0.223	0.224	-

References

[1] Haynes, D. R. L., William M. & Thomas J., B. *CRC Handbook of Chemistry and Physics: A Ready-reference Book of Chemical and Physical Data* (CRC Press, Boca Raton, Florida, 2016).

[2] James Gareth, T. H., Daniela Witten & Tibshirani., R. *An Introduction to Statistical Learning. 8st ed.* (Springer Texts in Statistics, New York, NY, 2017).

[3] Vittinghoff, G. D. V. S. S. C., E. & McCulloch, C. E. *Regression Methods in Biostatistics Linear, Logistic, Survival, and Repeated Measures Models.* (Springer New York, New York, NY, 2012).
